# A Novel Category of Anti-Hypertensive Drugs for Treating Salt-Sensitive Hypertension on the Basis of a New Development Concept

**DOI:** 10.3390/ph3010059

**Published:** 2010-01-07

**Authors:** Makoto Katori, Masataka Majima

**Affiliations:** Department of Pharmacology, School of Medicine, Kitasato University, Sagamihara, Kanagawa 228-8555, Japan

**Keywords:** renal kallikrein, ATP-sensitive potassium channel, poststatin ebelactone B, salt-sensitive hypertension

## Abstract

Terrestrial animals must conserve water and NaCl to survive dry environments. The kidney reabsorbs 95% of the sodium filtered from the glomeruli before sodium reaches the distal connecting tubules. Excess sodium intake requires the renal kallikrein-kinin system for additional excretion. Renal kallikrein is secreted from the distal connecting tubule cells of the kidney, and its substrates, low molecular kininogen, from the principal cells of the cortical collecting ducts (CD). Formed kinins inhibit reabsorption of NaCl through bradykinin (BK)-B_2 _receptors, localized along the CD. Degradation pathway of BK by kinin-destroying enzymes in urine differs completely from that in plasma, so that ACE inhibitors are ineffective. Urinary BK is destroyed mainly by a carboxypeptidase-Y-like exopeptidase (CPY) and partly by a neutral endopeptidase (NEP). Inhibitors of CPY and NEP, ebelactone B and poststatin, respectively, were found. Renal kallikrein secretion is accelerated by potassium and ATP-sensitive potassium (K_ATP_) channel blockers, such as PNU-37883A. Ebelactone B prevents DOCA-salt hypertension in rats. Only high salt intake causes hypertension in animals deficient in BK-B_2 _receptors, tissue kallikrein, or kininogen. Hypertensive patients, and spontaneously hypertensive rats, excrete less kallikrein than normal subjects, irrespective of races, and become salt-sensitive. Ebelactone B, poststatin, and K_ATP_ channel blockers could become novel antihypertensive drugs by increase in urinary kinin levels. Roles of kinin in cardiovascular diseases were discussed.

## Contents

1. Introduction

2. What is the renal kallikrein-kinin system (renal KKS)? Is it equipped to rectify excess sodium intake?

2.1. Renal kallikrein and low molecular weight kininogen

2.2. Kallikrein inhibitors

2.3. Kinins and kinin receptors

2.4. Renal kininases and their selective inhibitors

2.5 Stimuli for renal kallikrein secretion in the kidney

2.5.1. Sodium

2.5.2. Sodium-retaining steroid hormones

2.5.3. Potassium

2.5.4. ATP-sensitive potassium channel blockers

2.6. Roles of the renal KKS in the kidney

2.6.1. Vasodilating action of bradykinin in the kidney

2.6.2. Diuresis and natriuresis

3. Are animals deficient in components of the renal KKS hypertensive? — Additional high sodium intake is necessary. 

3.1. BK-B2 receptor-gene-disrupted mice 

3.2. Tissue kallikrein-gene-disrupted mice

3.3. Kininogen-deficient rats

4. How does excess sodium intake cause hypertension?

4.1. Kininogen-deficient BN-Ka rats are salt-sensitive.

4.2. Sodium accumulation in cells, particularly in blood vessels.

4.3. Increased sensitivity of vascular smooth muscles as a result of sodium accumulation.

4.4. Sodium accumulation in the cerebrospinal fluid and increased sympathetic discharge.

5. Do hypertensive patients secrete less urinary kallikrein?

5.1. Hypertensive patients.

5.2. Salt-sensitive hypertension.

5.3. Hypertensive animals.

6. Anti-hypertensive effects of urinary kininase inhibitors and renal kallikrein releasers in salt-induced hypertension models.

6.1. Agents that accelerate release of renal kallikrein.

6.2. Inhibitors of renal kinin-destroying enzymes. 

7. Roles of KKS on cardiovascular disorders and angiogenesis and a therapeutic aspect of tissue kallikrein-gene delivery.

7.1. Cardiac ischemia and ACE inhibitors

7.2. Ischemic preconditioning and reperfusion of the heart

7.3. Angiogenesis

7.4. Tissue kallikrein-gene delivery

8. Proposal of a new category of anti-hypertensive drugs on the salt-sensitive hypertension.

## 1. Introduction

The mechanisms of development of hypertension have not been fully identified, but the following important types of antihypertensive drugs have been widely used: Diuretics (inhibitors of sodium–potassium-chloride co-transporters in the thick ascending limb of Henle’s loop and of sodium-chloride cotransporters in the distal convoluted tubules, and other diuretics), calcium channel blockers, and inhibitors of the renin-angiotensin-system, which include angiotensin converting enzyme (ACE) inhibitors, angiotensin receptor antagonists, and aldosterone receptor antagonists and others. Use of these drugs as anti-hypertensives appears to offer satisfactory control of hypertension. However, we would like to propose a new group of the anti-hypertensive drugs on the basis of a novel concept of the mechanisms whereby salt-sensitive hypertension develops. 

It is known that seawater fishes avoid dehydration by the surrounding seawater by excreting NaCl mainly from their gills, while fresh water fishes combat over-hydration from the surrounding fresh water by absorbing NaCl and other ions from the gills, intestine, and kidney [1,2,3]. Terrestrial animals must conserve water and NaCl to survive in dry environments [1], and so the kidney has to reabsorb as much water and sodium as possible, so that 95% of the sodium filtered from the glomeruli is reabsorbed before it reaches the distal connecting tubules of the kidney. The tubuloglomerular feedback system in the kidney works at a maximum rate all the time to prevent the loss of essential water and sodium. The rest of sodium in the distal tubules may be reabsorbed in the collecting ducts (CD). So what would happen should a human ingest excess sodium? Are the kidneys equipped with a compensating system? The answer is yes. We believe that the renal kallikrein-kinin system (renal KKS) performs this role by generating kinins along the distal tubular systems, which inhibit NaCl reabsorption along the CD. As described below, salt-sensitive patients excrete low levels of urinary kallikrein. Thus, in order to treat patients with salt-sensitive hypertension, it is essential to accelerate secretion of renal kallikrein and/or to inhibit degradation of active bradykinin along the CD. As discuss in more detail, below, we have useful drugs for achieving this by interacting with the renal KKS. The rationale will be discussed by presenting persuasive supporting evidences in the following sections. 

## 2. What Is the Renal Kallikrein-Kinin System (Renal KKS)? Is it Equipped for the Rectification of Excess Sodium Intake?

### 2.1. Renal Kallikrein and Low-Molecular-Weight Kininogen

Kallikrein was first discovered in human urine as a substance that can reduce dogs’ blood pressure by intravenous injection in 1925 [4]. At first it was thought that this substance originated in the pancreas and it was thus named ‘kallikrein’, from *‘kallikreas’*, which means ‘pancreas’ in Greek [5]. Later, kallikrein was recognized as an enzyme that releases kallidin (Lys-bradykinin) from precursor substances in the plasma, and it is known that kallidin is the essential hypotensive substance. Since then, the eyes of the researchers have been focused on substances which act on the systemic blood pressure, either hypotensive or hypertensive, but this has happened to the detriment of interest in urine, whose importance has been largely overlooked by researchers. M. Rocha e Silva was looking for substances derived from the venom of the snake *Bothrops jararaca* which reduce the systemic blood pressure. Finally, in 1949 he discovered a nonapeptide (see Figure 2) and named it bradykinin (BK), since BK is not only hypotensive, but also slowly (*brady* in Greek) contracts (*kinein* in Greek) guinea pig ileum [6]. Now, Met-Lys-BK, Lys-BK (kallidin), and BK are found in plasma and other biological fluids and they are collectively called kinins. The biological actions of BK, the most popular nonapeptide, are reported to be potent vasodilatation, increased vascular permeability, smooth muscle contraction, pain sensation, natriuresis and renal blood flow increase. 

As is well known, the kallikrein-kinin system (KKS) is nearly analogous to the renin-angiotensin system (RAS), since kallikrein, a serine protease, generates kinin, an active substance, from the precursor proteins, kininogens. Importantly, there are two KKSs in the body, plasma KKS and tissue (or glandular) KKS. It is well known that angiotensin-converting enzyme (ACE) inactivates BK. Tissue kallikreins are different from plasma kallikrein. The former are secreted from the glandular tissues and the kidney. Tissue kallikrein generates kallidin (lysyl-BK) from low-molecular weight (LMW) kininogen, which is generated from hepatic cells. Plasma kallikrein is present in plasma in an inactive form, prekallikrein, which is activated by the activation of coagulation factor XII. Active plasma kallikrein releases bradykinin from high molecular weight (HMW) kininogen in plasma. The whole system and its functions are discussed in other review articles [7,8,9].

It is obvious that the plasma KKS in plasma does not play a major role in preventing hypertension. In fact, intraperitoneal injection of captopril, an ACE inhibitor, to anesthetized rats slightly increases the BK level in the arterial blood up to 29 pg/mL, but this increase is not sufficient to reduce the systemic blood pressure (BP), since intravenous infusion of nearly 1000 ng/min (about 1000 pg/mL in the arterial blood) is required to decrease the systemic blood pressure [10]. Thus, the increase induced in the BK concentrations in the arterial blood by ACE inhibitors is not sufficient to reduce the systemic BP.

**Figure 1:**
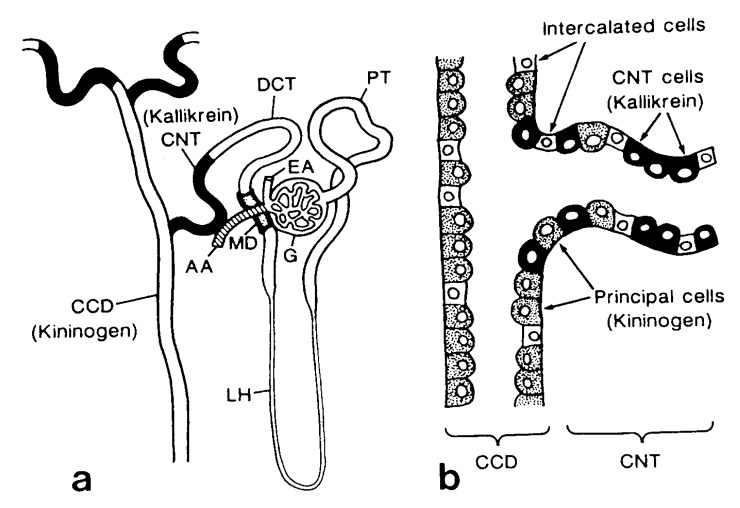
(a) Diagram of the immunocytochemical localization of kallikrein and kininogen in the human nephron and (b) a schematic representation of the intermingled CNT cells and principal cells at the junction between CNT and CCD. AA, afferent arteriole; CCD, cortical collecting duct; CNT, connecting tubule; DCT, distal convoluted tubules; EA, efferent arteriole; G, glomerulus; MD, macula densa; LH, Loop of Henle: PT, proximal tubule. (reproduced from Ref. [13] with permission).

The renal KKS, one of the tissue KKSs, works independently from the plasma KKS. Renal kallikrein (KLK_1_), a serine protease, is secreted from the cells in the connecting tubules (CNT) in the distal nephrons. The location of the kallikrein-secreting cells next to the distal convoluted tubule cells (Figure 1a) [11,12,13] is critical, since 95% of the filtered sodium is reabsorbed through the proximal nephrons as far as the distal convoluted tubule cells, and the CNT cells are located distal to these major reabsorption sites. A substrate of renal kallikrein, low molecular weight kininogen, is also secreted simultaneously from the principal cells of the cortical collecting ducts (CD), which coexist side by side with the CNT cells in the same transitional tubules [13] (Figure 1b). The formed kinins inhibit reabsorption of NaCl through the bradykinin receptors (BK-B_2_) distributed along the collecting tubules. It was also reported by a stop-flow method study of the nephron that renal kallikrein was secreted in the distal nephron and showed peak values of 1-2 mL after a peak of potassium [14].

The importance of the secretion sites of renal kallikrein in the distal nephron is shown in the following experiments. Furosemide is well known to exert diuretic action by inhibiting Na^+^-2Cl^-^-K^+^ co-transporters in the ascending limb of Henle’s loop. It is interesting that the initial part of its diuretic action is attributable to the renal KKS. Pretreatment of rats with FR17365 (8[3-[N-[(E)-3-(6-acetamidopyridin-3-yl)acryloylglycyl]-N-methylamino]-2,6-dichlorobenzyloxyl]-2- methylquinoline), a BK-B_2_ receptor antagonist, suppresses the initial phase of increase in urine volume and in the concentrations of sodium and chloride, but not that of potassium, which is induced by furosemide [15]. This indicates that the initial rapid phase of the diuretic action of furosemide may be attributed to renal kallikrein probably secreted, as result of a potassium overflow into the connecting tubules. The kinins formed inhibit reabsorption of NaCl through the bradykinin receptors (BK-B_2_) along the collecting tubules.

### 2.2. Kallikrein Inhibitors

Renal kallikrein, once released, is inactivated by a tissue kallikrein inhibitor, kallistatin, after release of kinins into the lumen of the cortical CD of the kidney. Kallistatin is synthesized mainly in the liver, but also, at lower expression levels, in the kidney [16]. The mRNA of kallikrein-binding-protein (KBP), an analogue of human kallistatin, was detected most abundantly in the inner medullary CD, together with only small amounts (about 1/10) in the outer medullary CD, the proximal convoluted tubules and the glomeruli, but no signals were found in the CNT or the cortical CD [17]. 

### 2.3. Kinins and Kinin Receptors

In human urine, large amounts of BK, kallidin and methionyl-lysyl-BK were found [18,19,20]. Rat urine also contains kinins, which must be generated in the kidney and in the urine itself, since infusion of kallikrein did not increase the excretion rate of kinins in man [21,22], although kallikrein generates kinins in plasma. Infusion of BK in into a human subject or into the renal artery of the dog also did not raise the urinary kinin levels [22,23], because of the very rapid degradation of kinins in the proximal tubules. 

In the isolated nephron segment of the rabbit, the [3H] BK binding capacity is maximal in the cortical CD and the outer medullary CD, while it is small but significant in the glomeruli, proximal straight tubules, cortical thick ascending limbs of Henle’s loop, and distal convoluted tubules [24]. These results are in conformity with the significant inhibition of the tubular efflux of ^23^Na in the distal nephron segments by BK administration into the lumen of late proximal convoluted tubules [25]. BK-B_2_ receptors were cloned [26] and were found to be G protein-coupled protein. The results using chemically cross-linked conjugates of bovine serum albumin with a BK-B_2_ agonist and using the potent BK-B_2_ antagonist, HOE 140, differ slightly from each other. Their binding sites were found not only in the CNT and the CD, but also in straight portions of the proximal tubules, in the thick ascending limb of Henle’s loop of the rat kidney [27]. The BK-B_2_ receptors are colocalized in the CNT cell layers with kallikrein and in the CD cell layers with kininogens [27]. The BK-B_2_ receptor mRNA is colocalized with kininogen mRNA in the kidney, and the most intense signals are observed in the distal tubules and CDs [28]. The localization of BK-B_2_ receptors in the CD may be compatible with the observations that BK inhibits the net sodium absorption in the CD [29]. In an established line of principal cells of the rabbit CD, BK stimulated cytosolic calcium and inositol phosphate formation in a dose-dependent manner from 1 nM to 1 μM and inhibited the arginine vasopressin-dependent increase of cyclic adenosine monophosphate [30]. 

BK activates a transient Cl^-^ selective (anion) secretion and stimulates basal to apical Cl^-^ secretion in the isolated mouse inner medullary CD epithelium. These currents are consistent with bradykinin activation of a Ca^2+^-dependent Cl^-^ conductance and expression of a mRNA transcript with 96% identity to mCLCA1/2 was confirmed [31]. In tubular fluid samples obtained from the medullary collecting ducts of Dahl/Rapp salt-resistant rats, the BK-B_2_ receptor antagonist, HOE 140, enhances Cl^-^ and water absorption [32].

### 2.4. Renal Kininases and Their Selective Inhibitors

**Figure 2:**
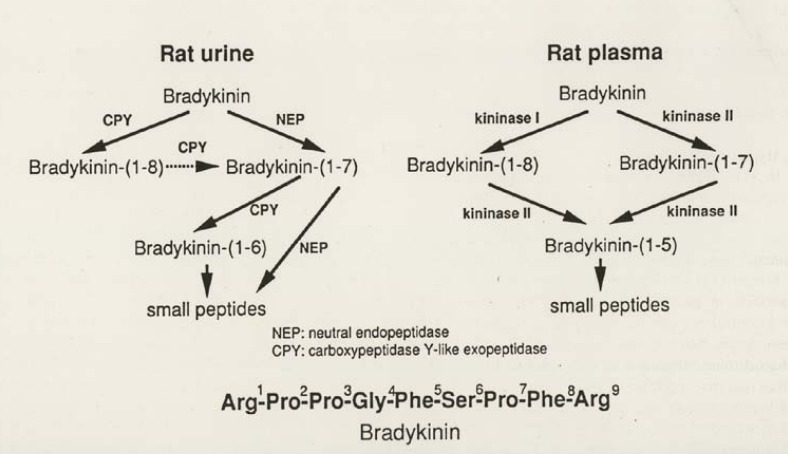
Pathways of bradykinin (BK) degradation by rat plasma and rat urine. BK(1-n) indicates BK degradation products with n amino acids from the *N*-terminal (reproduced from Ref. [9], with permission).

It is most characteristic that the degradation pathway of BK by kininases (kinin-destroying enzymes) in the collecting tubules of the kidney and in urine is completely different from that in plasma. Degradation of BK in urine is not inhibited by ACE inhibitors. As shown in Figure 2, we analyzed the pathway of BK degradation by rat and human urine and found that the major kininases in human and rat urine are carboxypeptidase-Y-like exopeptidase (CPY) and neutral endopeptidase (NEP) [33]. Carboxypeptidase Y was originally found in yeast. This enzyme activity in rat urine was identified by the inhibitor spectrum and an antibody against a peptide fragment, but unfortunately the total structure could not be determined because of the small quantity of rat urine, so it is tentatively designated carboxypeptidase Y-like exopeptidase (CPY) [33]. 

NEP has been reported to be present mainly in the proximal tubules, using the isolated nephron segments [34], by immunocytochemistry [35], and by the stop-flow method [36]. It was reported that NEP accounts for 35–59% of its total kininase activity, while kininase I and kininase II, originally present in the plasma, account for 11–13% and 29–44% [37]. It is important that the NEP enzyme activity is entirely dependent on its pH, and that the NEP activity is at its maximum at neutral pH. Erdös’s group reported a human urinary carboxypeptidase distinct from carboxypeptidases A, B, or N [38], but the new carboxypeptidase was not identified. Carboxypeptidase M was also detected in human urine [39], and prolyl endopeptidase (or postproline cleaving enzyme) was reported in the kidney tissue [40].

CPY and NEP in urine are not inhibited by the ACE inhibitors. We found two urinary kininase inhibitors, ebelactone B [41] and poststatin [42]. Ebelactone B was originally isolated from the culture medium of *Actinomysetes* as an esterase inhibitor [43]. Its structure is 3,11-dihydroxy- 2,4,6,8,10,12-hexamethyl-9-oxo-6-tetradecenoic 1,3-lactone [44]. Poststatin, which is isolated from a fermentation broth of *Streptomyces viridochromogenes* as an inhibitor of prolyl endopeptidase**[45] has the structure L-valyl-L-valyl-3-amino-2-oxovaleryl-D-leucyl-L-valine [46]. Ebelactone B was recently reported to exhibit selective inhibition of yeast carboxypeptidase Y in a mixed non-competitive manner [47].

Ebelactone B exerts its inhibitory action on CPY in urine [41,48] and poststatin inhibits both CPY and NEP in urine [42]. Intra-duodenal administration of ebelactone B (3 mg/kg) to anesthetized rats caused marked diuresis (by 110%) and natriuresis (by 110%), in parallel with the increase in urinary kinin levels (by 110%). Urinary potassium levels were kept fairly constant. Intravenous infusion of a BK-B_2_ antagonist, HOE 140 (3 mg/kg/hr), strongly blocked both ebelactone B-induced diuresis and natriuresis [48]. 

Additionally, *in vivo* transfer of antisense oligonucleotide against CPY kininase inhibited urinary CPY kininase activity and also significantly reduced the rise of systolic blood pressure (SBP) in DOCA-salt hypertension of SD-rats, together with urinary reduction of sodium excretion and urinary CPY kininase activity in urine [49]. The inhibitory effects of ebelactone-B and poststatin in pathological states will be further described in Section 6.2. 

### 2.5. Stimuli for Kallikrein Secretion in the Kidney

#### 2.5.1. Sodium

In human subjects, it was reported that a relationship was observed between kallikrein and sodium in urine [50]. However, the further clinical studies revealed no direct correlation between urinary sodium and kallikrein excretion in a large population of normal adults [51] or in hypertensive adults [52]. A positive correlation between urinary kallikrein and sodium was also not found in over 600 normal children over 5-year period [53]. This inconsistency may have arisen because the subjects were free to choose their diets, and there was no restriction on the contents of sodium and potassium or the volume of fluid that could be consumed. Thus, the effects of high sodium intake on urinary kallikrein excretion are controversial.

In contrast, sodium restriction, rather than sodium loading, accelerates the excretion of renal kallikrein. In normal human subjects, intravenous water loading during prolonged sodium restriction produced a significant increase in kallikrein excretion, but not during the period of normal sodium intake [54]. A low dietary sodium intake or sodium restriction has consistently been observed to increase urinary kallikrein excretion in human [51,54,55] and in rats [56,57]. In micro-dissected segments of rabbit nephrons [58], low sodium intake markedly increases the levels of both active and inactive kallikreins in the granular portion of the distal convoluted tubules and in the cortical CD (or CNT) without altering either the distribution profile or the ratio of active- to total-kallikrein in the nephron or the urine. 

#### 2.5.2. Sodium-Retaining Steroid Hormones

Prolonged sodium deprivation caused aldosterone release through activation of the renin-angiotensin system. Thus, the increase in kallikrein excretion may occur together with the release of this hormone. In fact, a large accumulation of data indicates a positive correlation between the activity of sodium-retaining steroid hormone and renal kallikrein excretion. Urinary excretion of kallikrein is increased in patients with primary aldosteronism [59], in normal volunteers of patients with essential hypertension on a diet of low sodium or high potassium [51], after treatment with 9-fluorohydrocortisone [50], and in Bartter’s syndrome [60]. Treatment of patients with primary aldosteronism and treatment of normal volunteers with spironolactone, a selective antagonist of aldosterone, markedly reduced urinary kallikrein excretion [51,61]. Removal of aldosterone-producing tumors also reverses the increased excretion of urinary kallikrein [62]. Administration of aldosterone enhances urinary excretion of potassium. Moreover it is possible that increased intraluminal concentrations of potassium in the kidney, induced by aldosterone, accelerate kallikrein excretion. 

#### 2.5.3. Potassium

An electron-microscope study [63] reveals that a high-potassium diet produces hypertrophy and hyperplasia of the kallikrein-containing cells of rats, including hypertrophy of the components of the Golgi complex and of the rough endoplasmic reticulum, and a large number of secretory-type vesicles containing kallikrein, suggesting that a high-potassium diet increases the synthesis and secretion of renal kallikrein. Furthermore, it is reported [64] that potassium supplement induced 70 and 40% increases in urinary kallikrein levels and renal BK-B_2_ receptor density respectively, but did not change serum kininogen levels. Northern blot analysis showed that renal kallikrein mRNA levels increased 2.7-fold and competitive RT-PCR (reverse transcription-polymerase chain reaction) showed a 1.7-fold increase in renal bradykinin B_2_ receptor mRNA in SHR.

These results were confirmed in *in vivo* animal experiments and in kidney slices. Intravenous infusion (6.0 mL/kg/h) of high potassium solution (67.5 mM KCl+67.5 mM NaCl) to anesthetized rats induced a rapid increase of kallikrein (by 49%) and reached its maximum 30 min after the start of the K^+^ infusion. The urine volume and the urinary excretion of Na^+^ were increased (by 47.6 % and 32.2% in total, respectively) 60 min after the start in parallel with an increase in the excretion of K^+^ and Cl^-^[65]. The diuresis and natriuresis induced by high potassium were almost completely suppressed by a BK-B_2_ antagonist, FR173657, indicating that diuresis and natriuresis by high-potassium infusion are attributable to renal kallikrein excretion. Intravenous infusion of high-potassium saline (K^+^ 75 mM + Na^+^ 75 mM) to rats also induced rapid excretion of the renal kallikrein, which was augmented within 15 to 30 min, whereas infusion of physiological saline (150 mM NaCl) at the same speed did not significantly increase the kallikrein excretion [66]. The rapid excretion could not be explained by aldosterone release, since aldosterone release by intravenous infusion of potassium takes at least 1 hr in humans [67]. 

This rapid increase of kallikrein was further confirmed by *in vitro* experiments. Sliced cortex isolated from rat kidney was superfused with an isotonic solution containing 4 to 75 mM KCl. Twenty to 70 mM KCl solution dose-dependently enhanced secretion of renal kallikrein [68]. The addition of a physiological concentration (4 mM) of potassium into the superfusion fluid did not affect the release of renal kallikrein.

In the dissected renal connecting tubules of Sprague-Dawley rats (4-6 weeks old), the maximum effect of potassium on kallikrein secretion was observed 10 min after incubating the tubules with buffer containing 20 mM potassium [69]. 

#### 2.5.4. ATP-Sensitive Potassium Channel Blockers

It is thought that the rapid secretion of renal kallikrein caused by potassium is mediated by the inhibition of the potassium channels of the CNT cells. Glibenclamide, an insulin releaser from the pancreatic β-cells, is reported to be an ATP-sensitive potassium (K_ATP_) channel blocker and to inhibit the transporter of Na^+^ and K^+^ channels, thus preventing kaliuresis [70]. Intravenous injection of glibenclamide (1 to 30 mg/kg) during physiological saline infusion in anesthetized rats causes a dose-dependent increase in urinary kallikrein [66]. Despite the administration of glibenclamide, rats showed slightly (but insignificantly), lower blood glucose levels (0.3% NaCl: 177 ± 13 mg/100 mL; glibenclamide 30 mg/kg daily: 159 ± 4 mg/100 mL; glibenclamide 60 mg/kg daily: 160 ± 8 mg/100 mL) than that of normal control (0.3% NaCl intake) and no rats died during the experiments [71]. In rats, this may have been attributable to the constant, *ad libitum*, dietary intake. 

A kidney-selective ATP-sensitive potassium channel blocker, U18177 (*N,N’*-dicyclo- hexyl-4-morpholinecarboxamidine) and its l-adamantyl analog, 4-morpholinecarboximidine- *N*-1-adamantyl-*N’*-cyclohexylhydrochloride (PNU-37883A, Pharmacia, Upjohn) were initially developed as an orally effective nonkaliuretic diuretic in rats [72]. PNU-37883A required a 60-fold higher dose of hydrochlorothiazide and furosemide for natriuretic activity, but showed a more prolonged natriuretic effect (4-6 hours), than hydrochlorothiazide and furosemide [72]. PNU-37883A inhibited relaxation of rabbit mesenteric artery induced by four potassium channel openers, cromakalim, minoxidil sulfate, pinacidil, and RP-49356 [73]. In an insulinoma cell line, PNU-37883A, unlike glibenclamide, failed to inhibit K_ATP_ current [74]. It was observed [75], that microperfusion of the loop of Henle and the cortical distal tubule, and also by patch-clamp technique, that PNU-37883A disclosed the presence of K_ATP_ channels at the above two sites in the nephron. Thus, PNU-37883A inhibits 1) recycling of potassium across the apical membrane of the cells of the thick ascending limb of Henle, causing inhibition of the reabsorption of sodium and chloride by the Na^+^/2Cl^-^/K^+^-cotransport mechanism and 2) potassium channels in the apical membrane of the principal tubule cells of the cortical collecting tubule, causing inhibition of potassium secretion. However, the dose of PNU37883A used for the microperfusion in the experiments was 50 μM, which is 50-5,000 times higher than that used for the release of renal kallikrein from the superfused slice of kidney cortex of rats, as mentioned below. The K_ATP_ channels in the CNT cells may be much more sensitive to PNU-37883A than that of the principal cells of cortical CD.

The administration of PNU-37883A (10 mg/kg) to Sprague-Dawley rats also brought about increased urinary excretion of kallikrein together with increases in urine volume and sodium excretion [66]. PNU-37883A had no additive effect on the increase in renal kallikrein excretion induced by high potassium, suggesting that high potassium also may exert the same cellular actions as the K_ATP_ channel blockers by inhibition of potassium efflux and depolarization of the CNT cells. In fact, it was reported [76] that perfusion of the inner medullary collecting tubules with high concentration of potassium causes depolarization of the tubular cells. Perfusion with barium chloride, a potassium channel blocker, also exerts the same effect. Renal potassium channels in the distal nephrons not only secretes potassium, but also maintains the membrane potential of the tubular cells [77].

**Figure 3:**
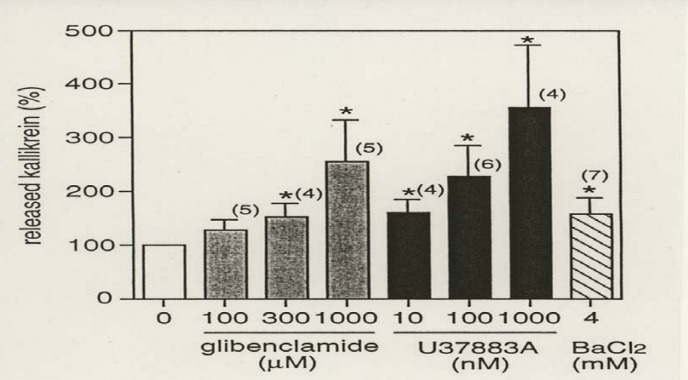
Effects of ATP-sensitive potassium channel blockers on the release of kallikrein from sliced cortex of rat kidney. A slice of kidney cortex was isolated from Sprague-Dawley strain rats and superfused with 10 mM HEPES (pH 7.4) +140 mM NaCl+1 mM CaCl_2_ at a flow rate of 0.14 ml/min for 60 min. Then each ATP-sensitive potassium channel blockers, glibenclamide (shaded columns), U37883A (closed columns), and barium chloride (hatched column), was added into the solution and the superfused solution was collected. Values are mean ±S.E.M. Numbers of rat used are indicated in parenthesis. * P < 0.05, compared with the solution without the blockers (open column) by Student’s t-test (reproduced from Ref. [68] with permission).

The above observation was also confirmed by *in vitro* experiments. Glibenclamide (0.1 to 1 mM) and PNU-37883A (0.01 to 1 μM) induced a concentration–dependent release of renal kallikrein from the slices of rat kidney cortex [68] (Figure 3). The K_ATP_ channels of the cortical CD in the kidney may be slightly different from the β-cells of the pancreas, because PNU-37883A is selective to the CNT cells and 1,000 times more potent than glibenclamide. Barium chloride, which inhibits intracellular potassium efflux by blocking the channels, also showed increase in the kallikrein release. The renal kallikrein release from the slice by high potassium concentrations or K_ATP_ channel blockers was reduced by the absence of calcium or in the presence of a voltage-dependent calcium channel blocker, nifedipine, in the superfused solution [68]. The renal kallikrein release was inhibited by cytochalasin B, indicating that the exocytosis of renal kallikrein is induced by polymerization of actins. 

In dissected renal connecting tubules of young Sprague-Dawley (SD) rats (4-6 weeks old), kallikrein secretion was increased in a concentration-dependent manner by the kidney-specific K_ATP_ channel blocker, PNU-37883A (0.1, 1, 10, and 100 μM)[69].

The CNT cells participate, at least in part, in the secretion of potassium in the distal nephrons [78,79]. The cortical CD is known to be the major site of potassium secretion in the nephron. The cells lining the cortical CD can be divided into at least three cell types: CD cells and alpha- and beta-intercalated cells. The CD cells are characterized by the presence of Na^+^-K^+^ pump and K^+^ channels in the basolateral membrane, and amiloride-sensitive Na^+^ channels and low conductance K^+^ channels in the apical membrane. These transport properties are compatible with the K^+^-secretory function. Because the CNT cells share similar transport properties with the CD cells, it is reasonable to assume that the CNT cells may also participate in K^+^ secretion. The apical low-conductance K^+^ channel, which is critical for K^+^ secretion into the lumen, is a member of the ATP-sensitive K^+^ channel and is blocked by Ba^2+^[80]. Thus, it is likely that renal kallikrein secretion from CNT cells is linked to inhibition of this low-conductance K^+^ channel that maintains the membrane potential of tubular cells. Because high potassium concentrations and administration of BaCl_2_ in the tubular lumen caused depolarization of the cell membrane voltage, changes in the membrane voltage of the CNT cells may be one of the factors responsible for kallikrein secretion.

Taken together, these results suggest that the application of high potassium and the inhibition of the K_ATP_ channels cause depolarization of the CNT cells and induce exocytosis of renal kallikrein by calcium influx via voltage-dependent calcium channels.

### 2.6. Roles of the Renal KKS in the Kidney

#### 2.6.1. Vasodilating Action of BK in the Kidney

Many reviews on the physiological and pathological roles of the renal KKS, particularly in relation to hypertension, have been published [7,81,82,83,84,85,86,87]. As the roles of the renal KKS in the kidney were also reviewed by us [88], the most important concerns will be introduced here. A large number of studies have been published on these effects, since kallikrein and kinins were generally recognized as vasodilating and hypotensive substances.

Intravenous or intra-arterial administration of BK or kallidin induced renal arteriolar vasodilatation in normal subjects [89,90] and in anesthetized dogs [91,92,93]. Vasodilatation was observed in isolated blood-perfused canine kidneys and was partly attributable to prostaglandin generated by BK [94].

It was reported [95] that systemic administration of an ACE inhibitor, SQ 20881, induced a significant increase in renal blood flow in dogs, together with a reduction of the mean aortic pressure and a slight increase in urinary kinin excretion and renal venous kinin concentration. However, renal hemodynamic [96] in normal animals was not altered by the BK-B_2_ antagonists, HOE 140, or D-Arg^0^[Hyp^3^,Thi^5,8^, D-Phe^7^]BK (Thi^5,8^-BK) [96,97,98,99]. In the same way, in normotensive rats, increased renal blood flow caused by the ACE inhibitor, captopril, was not altered by treatment with Thi^5.8^-BK [100]. In dogs, Thi^5,8^-BK partially attenuated the increase in renal flood flow due to enalapril, an ACE inhibitor [98], but in rabbits it did not significantly attenuate such increases when they were induced by captopril or another ACE inhibitor, lisinopril [101,102]. HOE 140 also did not alter renal blood flow increases caused by ramiprilat or captopril in the rabbit [103] or by ramiprilat in the rat [104]. Thus, endogenous kinins do not appear to play an active role in normal or euvolemic rats.

In contrast, in hydropenic normotensive rats, renal blood flow increased by enalaprilat was decreased to a level similar to the pre-enalaprilat baseline by Thi^5,8^-BK, suggesting the contribution of kinins to hemodynamic changes in the hydropenic state [104].

As is well known, three regions of the kidney, the cortex, the outer and inner stripes of the outer medulla, and the inner medulla are supplied and drained by a specialized and independent vascular system [105]. An elegant study using laser-Doppler flowmetry, in which optical fibers were implanted into the renal cortex or the renal medulla, or both, determined the renal medullary blood flow changes separately from the cortical blood flow in anesthetized and conscious rats [106]. Infusion of BK (0.1 mg/min) to the renal medullary interstitium increased renal papillary blood flow to 117% without altering cortical blood flow or blood pressure in anesthetized Munich-Wistar rats [107]. The increased papillary flow was accompanied by a twofold increase in urine flow, sodium excretion, and fractional sodium excretion. Interstitial infusion of captopril to block the degradation of endogenous BK in the rat also increased the papillary blood flow by 21% without altering the cortical blood flow, indicating that endogenous BK may increase the papillary blood flow. The increase in the papillary blood flow induced by either BK or captopril was eliminated by pretreatment with L-NG-nitro-l-arginine-methyl ester (L-NAME), which inhibited nitric oxide generation. A BK-B_2_ receptor antagonist, Thi^5,8^-BK, reduced basal blood flow by 15% and blocked the rise in the papillary blood flow produced by captopril [108]. Renal prostaglandins moderated and mediated the actions of the renal KKS [81]. Thus, the renal cortical blood flow is basically separable from that of the renal medullary and papillary blood flow.

An increase in the papillary blood flow could be associated with increases in the urine flow and sodium excretion. Infusion of diltiazem, a calcium channel blocker, into the renal medullary interstitium of rats also selectively increased the papillary blood flow by 26% without altering the renal blood flow or the renal cortical blood flow [109]. Diltiazem caused a 70% rise in urine flow and sodium excretion and a 1.6-fold increase in fractional sodium excretion. In contrast, infusion of L-NAME into the renal medullary interstitium led to a decrease in papillary blood flow to 71% of the control with reduction of urine flow (by 39%) and sodium excretion (by 34%) without changes in fractional sodium and water excretion [110]. The observation that renal kallikrein in the granular cells of the CNT of the distal nephron is distributed in the luminal membranes and basolateral infoldings [11] may indicate that the renal kallikrein is secreted not only into the luminal side, but also into the interstitial space and kinins formed in the inner medullary CD may be distributed to the interstitial space to induce vasodilatation.

#### 2.6.2. Diuresis and Natriuresis

Injection of kinins intravenously or into the renal artery induced diuresis and natriuresis [89,111], and this increase was observed despite administration of anti-diuretic hormone (ADH) [112]. It was suggested that the natriuretic effect of kinins may be attributed either to the inhibition of sodium reabsorption in the distal part of the nephron or to a change in deep nephron reabsorption due to the change in the blood flow. Measurement of superficial cortical and papillary blood flows using laser-Doppler flowmetry revealed that enalapril (an ACE inhibitor) and phosphoramidon (a NEP inhibitor) along with 0.3 M sodium bicarbonate solution, which inhibit kinin degradation and enhance urinary kallikrein activity, increased papillary blood flow by 50% without altering the glomerular filtration rate or the outer cortical blood flow [97]. Probably the limited increase in blood flow may be attributable to the weak kininase activity of NEP in rat urine. The increase in papillary blood flow was returned to the control level by Arg^5,8^-BK, a kinin antagonist, suggesting that renal kallikrein secreted into the papillary interstitial space may be responsible for the increase in the papillary blood flow. However, the increase in urine flow and sodium excretion, caused by enalapril and phosphoramidon, was not altered by the BK antagonist [97]. The ineffective inhibition of urine flow and sodium excretion by phosphoramidon may be attributable to the limited contribution of NEP as a kininase in rat urine. Administration of Arg^5,8^-BK alone lowered papillary blood flow by 20%, without affecting outer cortical blood flow or the glomerular filtration rate. Urine flow decreased and urine osmolarity increased after the rats received the kinin antagonist, but sodium excretion remained unaltered [97]. 

In isolated perfused rat cortical CD, BK inhibited the net absorption of sodium and chloride without affecting net potassium transport, bicarbonate flux, or the transmembrane potential difference [29,113]. BK, administered systemically, is destroyed in the proximal tubules immediately after glomerular filtration, so that its diuretic and natriuretic actions are hard to demonstrate, but as discussed later, the diuretic and natriuretic actions of endogenous BK can be shown in the presence of renal kininase inhibitors and in their cancellation by BK-B_2_ antagonists.

## 3. Are Animals Deficient in the KKS Components Hypertensive?—Additional High Sodium Intake Is Necessary

### 3.1. BK B_2_ Receptor-Gene-Disrupted Mice

Mice that are homozygous for the targeted disruption of the gene encoding BK-B_2_ receptor (B_2_-KO) have been reported [114]. They lack responses in the ileum, uterus, and the superior cervical ganglion to BK. BK-B_1_- and B_2_-KO mice are normotensive, but exhibit a lower heart rate than controls [115]. In contrast, B_2_-KO mice have a greater pressure response to the chronic high sodium intake than controls do [116]. The mean blood pressure of B_2_-KO mice, maintained on a high Na^+^ diet (3.15% in food + 1% saline in drinking water) for eight weeks, was higher (114 ± 6 mmHg), than 79 ± 2.8 mmHg seen in B_2_-KO mice on a normal Na^+^ diet. Renal blood flow was reduced by 20% and renal vascular resistance was twice that of B_2_-KO mice on normal Na^+^. Control mice on high Na^+^ were normotensive and tended to have increased renal blood blow and decreased renal resistance, compared with control mice on a normal Na^+^ diet [117]. In another report [118], BK2R-/- mice showed a slightly higher blood pressure than wild-type BK2R(+/+) and BK2R(+/-) mice. Chronic salt loading (0.84 mmol/g chow for 15 days) increased the blood pressures of BK2R(-/-) and BK2R(+/-), whereas it did not affect BK2R(+/+) mice. 

BK-B_2_ receptors (B(2)R) null (-/-) and wild type (+/+) mice are fed normal (NS, 1% NaCl) or high (HS, 5% NaCl) salt diet during pregnancy [119]. After birth, the offspring remained with their mothers until they were weaned and were subsequently maintained on the respective maternal salt intake until they were four months of age. The age-related changes at three and four months in tail-cuff BP and anesthetized mean arterial pressure (MAP) at four months were not different in NS/B(2)R(-/-) and NS/B(2)R(+/+) mice. However, there was a mild increase in BP in NS/B(2)(-/-) at two months compared with that in NS/B(2)R(+/+). In contrast, HS/B(2)R(-/-) mice manifested early onset and persistent elevations of tail-cuff BP (P < 0.05) at two, three, and four months in comparison with other groups. MAP was also higher in HS/B(2)R(-/-) than HS/B(2)R(+/+), NS/B(2)R(-/-), and NS/B(2)R(+/+). Lack of B(2)R from early development does not alter the increase of BP when sodium intake was normal [119].

Although one report [120] stated that increasing dietary salt intake did not affect the mean arterial blood pressure or the heart rate of B_2_-KO mice, it is generally accepted that BK-B_2_ receptor-deficient mice are salt-sensitive.

The distribution of four different polymorphisms of the kinin B_1_ and B_2_ receptor genes was studied in a population of 20 normotensive and 77 hypertensive African-Americans [121]. Among the polymorphisms analyzed, a potentially and functionally significant polymorphism in the core promoter of the kinin B_2_ receptor (C-58-T transition) displayed greater prevalence of the C-58 allele in the hypertensive patients than in the control (0.72 *vs.* 0.62, P = 0.009). 

### 3.2. Tissue Kallikrein-Gene-Disrupted Mice

Mice that lack tissue kallikrein (TK) are unable to generate significant levels of kinins in most tissues or, as seen in B_2_-KO mice, and maintain normal blood pressure on a normal sodium diet, but develop cardiovascular abnormalities early in adulthood [122]. Tissue kallikrein deficient mice were studied as two kidney-one kidney clipped (2K1C) mice [123]. Blood pressure, monitored by telemetry and by plethysmography, rose immediately after clipping in both genotypes reached similar levels (2K1C-TK+/+: +24%, 2K1C-TK-/-: +21%). Tissue kallikrein-deficient mice show an absence of the 70-kDa form of γ-epithelial Na channels (γ-ENaC). In mice lacking the B_2_ receptor for kinins, the abundance of the 70-kDa form of γ-ENaC was increased, indicating that its absence in TK-/- mice is not kinin-mediated. In mouse cortical collecting ducts isolated and micro-perfused in vitro, the addition of TK to the luminal fluid significantly increased the intracellular Na^+^ concentration, indicating an activation of the luminal entry of the cation [124]. 

Nine single-nucleotide polymorphism in the human kallikrein gene were identified [125]. A significant decrease in urinary kallikrein activity was observed for the subjects who were heterozygous for the Arg53His polymorphisms, compared with other subjects. However, none of the polymorphisms was actively associated with hypertension (See details in Ref. [88]).

The rat strain of low kallikrein phenotype in urine was reported [126,127,128]. Kallikrein activity in kidney was reduced by 60% of normal Wister rats, but that in heart was increased and the tissue activities in the pancreas, liver, and submandibular glands were unchanged [128]. The SBP was slightly higher (130 mmHg *vs*. 114 mmHg in control) and increased to 153 mmHg after 10 days of dietary sodium loading [127]. 

### 3.3. Kininogen-Deficient Rats

**Figure 4:**
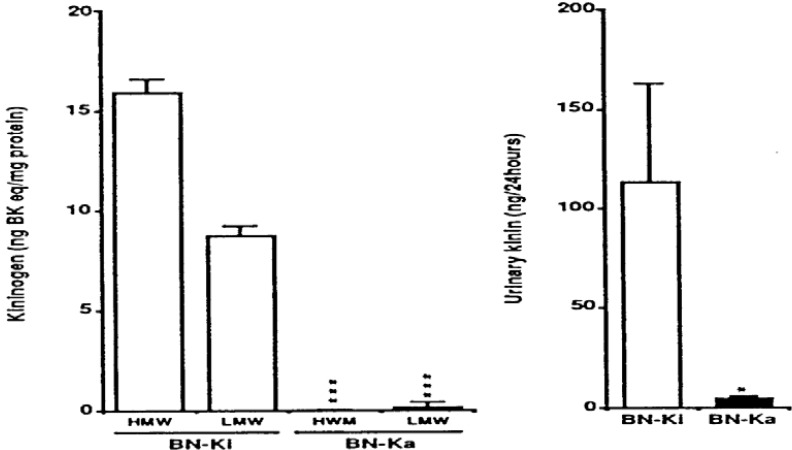
Kininogen levels in plasma (left panel) and excreted kinin levels in urine (right panel) in normal Brown Norway Kitasato (BN-Ki) rats and kininogen-deficient BN-Katholiek (BN-Ka) rats. Values are means ±S.E.M. of four rats. Values in BN-Ka rats were compared with those in BN-Ki rats. *P < 0.05, ***P < 0.001. BK eq, bradykinin equivalent; HMW, high molecular weight: LMW, low molecular weight (reproduced from Ref. [145] with permission).

Kininogen-gene-disrupted mice have not been reported, so we have been using mutant kininogen-deficient rats for further clarification of the roles of the renal KKS in relation to salt-sensitive hypertension. It was first reported that mutant rats of the BN strain (*Rattus norvegicus*, BN/fMai) are devoid of plasma kallikrein-like activity and show low levels of kininogen in plasma [129]. This work has been largely extended [130]. The deficient rats are termed Brown Norway-Katholiek (BN-Ka) rats [130], because they were originally reported by the Katholiek University of Leuven, Belgium. Further studies revealed that both HMW and LMW kininogens are almost absent from the plasma (Figure 4) [131,132], and that deficient BN-Ka rats are almost entirely incapable of excreting kinin in the urine (Figure 4) [132,133]. Normal rats of the same strain were kept at the Kitasato University animal facilities and are designated BN-Kitasato (BN-Ki) rats [130]. Normal BN-Ki rats show the same levels of kininogens as rats of other strains, such as the Sprague-Dawley (SD) strain [132]. The kininogen-deficient BN-Ka rats are capable of producing both kininogens in the liver. However, they cannot release kininogens into the bloodstream because of the point mutation of Ala^163^ to threonine in the common heavy chain in the structure of both kininogens [134]. These findings have been confirmed [135]. The mRNAs of HMW and LMW kininogens and prekallikrein that present in the liver of deficient BN-Ka rats are of similar size and abundance to those in control Brown Norway (BN-Orl) rats [136].

BN-Ka rats, in which kininogens are congenitally deficient in the plasma, have no apparent symptoms. Changes in systolic blood pressure (SBP) during growth in deficient BN-Ka rats are the same as those in normal BN-Ki rats, when they are fed on a diet containing 0.3% NaCl and drink distilled water [132]. Intra-arterial administration of angiotensin II or norepinephrine in conscious kininogen-deficient BN-Ka rats after infusion of 0.15 M (physiological) saline for 4 days was made. The dose-response curve of the increase in mean arterial pressure of deficient BN-Ka rats is not different from that for normal BN-Ki rats, suggesting that the arteriolar smooth muscle in deficient BN-Ka rats is no more sensitive to angiotensin II and norepinephrine than that of normal BN-Ki rats [137].

A congenital kininogen deficiency in the plasma was also reported in humans [138,139,140,141,142,143]. Activated partial thromboplastin time (aPTT) in these congenital patients was prolonged, but no SBP rise in these patients has been reported. 

Mutant kininogen-deficient BN-Ka rats responded with less plasma exudation in bradykinin-involved inflammatory models, such as in rat carrageenin-induced pleurisy and paw edema [131,144]. 

It is clear in the above discussion that deficiency of the components of renal KKS does not by itself induce hypertension. On the other hand, excess salt intake induces salt-sensitive hypertension in mice or rats with deficiencies in the components of KKS. How, then, is salt-sensitive hypertension induced? What mechanisms can be considered? Do we have antihypertensive agents against salt-sensitive hypertension? To answer these questions, kininogen-deficient rats, which do not excrete kinin in their urine (Figure 4), were extensively used to analyze the mechanisms. The detail will be precisely discussed in the following sections.

## 4. How Does an Excess Sodium Intake Cause Hypertension?

### 4.1. Kininogen-Deficient BN-Ka Rats Are Salt-Sensitive

**Figure 5:**
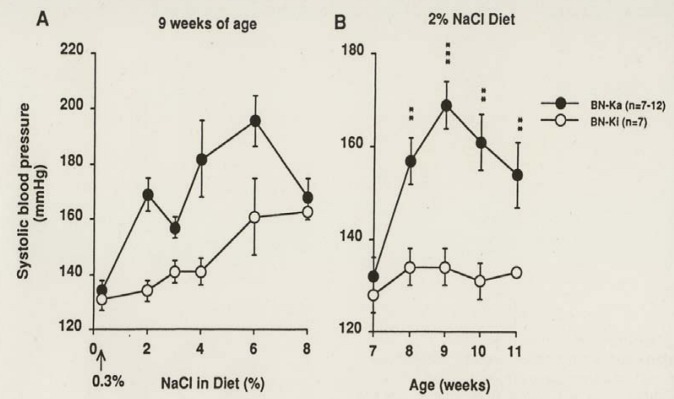
Changes in systolic blood pressure in normal Brown Norway-Kitasato (BN-Ki) rats and kininogen-deficient BN-Katholiek (BN-Ka) rats given NaCl-loaded diets. Both strains of rats were fed diet containing from 2% to 8% NaCl from the age of 7 weeks for two weeks (panel A) and a 2% NaCl diet between the ages of 7 to 11 weeks (Panel B). Values are means (±S.E.M.) of 7-12 rats. Values in BN-Ka rats were compared with those in BN-Ki rats of the same age. **P < 0.01, *** P < 0.001 (reproduced from Ref. [145] with permission).

Generally, a dietary concentration of more than 4% NaCl increases the systolic blood pressure (SBP) of normal strain rats. The same is true in normal BN-Ki rats. In contrast, kininogen-deficient BN-Ka rats showed an increase in SBP after receiving only 2% concentration of NaCl in their diet, as measured by tail cuff plethysmography [145] (Figure 5A). Figure 5B illustrates the changes in the SBP of rats of both strains fed on a 2% NaCl diet for four weeks. 

The kininogen-deficient BN-Ka rats increased the SBP to 167 ± 4 mmHg within two weeks, whereas the SBP of normal BN-Ki rats did not change during the 4-week period (134 ± 4 mmHg). During the period of feeding with a 2% NaCl diet, both strains of rats showed increases in water intake and urine volume, but deficient BN-Ka rats ingested more water and excreted less urine than do normal BN-Ki rats [145], so that the tentatively calculated difference (water intake minus urine volume) was much greater in the former than in the latter, which was constant during the 4-week period. Urinary excretion of sodium also increased, but kininogen-deficient BN-Ka rats excreted less than normal BN-Ki rats. Urinary excretion of potassium and creatinine did not differ between normal BN-Ki rats and deficient BN-Ka rats. Despite the reduced excretion of sodium and water in deficient BN-Ka rats, the serum levels increased slightly only at nine weeks of age, whereas those of normal BN-Ki rats were constant. Importantly, the sodium levels in the erythrocytes during the 2% sodium loading were increased significantly in the mutant BN-Ka rats but remained constant in the normal BN-Ki rats. Plasma renin activity was reduced and then tended to increase, but there was no difference between the two strains. A causal effect of the kininogen deficiency on the increased SBP was examined with a 7-day subcutaneous infusion of LMW kininogen administered from day 8 by a mini-osmotic pump, which was implanted subcutaneously in the back of kininogen-deficient BN-Ka rats fed a 2% NaCl diet [145]. The infusion lowered the SBP to the control level and caused increases in urinary kinin, sodium excretion, and urine volume. In contrast, subcutaneous infusion of HOE 140, a selective BK-B_2_ receptor antagonist, into normal BN-Ki rats, on 2% NaCl in diet, increased the SBP to 166 ± 3 mmHg and reduced excretion of urinary sodium and urine volume.

These results clearly indicate that kininogen-deficient BN-Ka rats have difficulty in excreting sodium and water and are extremely sensitive to ingested salt. In these rats, ingestion of 2% NaCl in the diet caused an accumulation of sodium in the erythrocytes, water retention in the body and subsequent hypertension, which are directly related to kininogen deficiency or lack of kinin generation in the urine. There is a report that the tentative SBP rise in kininogen-deficient BN-Ka rats induced by a high salt diet was not confirmed [135]. 

Salt-induced experimental hypertension is characterized by early increase in fluid volumes and cardiac output, but it is reported [146] that these parameters subsequently revert toward normal. The peripheral resistance initially decreases slightly and then continues to increase. Thus, the increased arterial BP is subsequently maintained by this elevated peripheral resistance. 

The intake of more than 4% of NaCl in the diet reduces the excretion of active urinary kallikrein, but not of urinary prokallikrein in both normal BN-Ki rats and deficient BN-Ka rats [145]. Thus, the increase in SBP in normal BN-Ki rats, which took diet containing over 4% NaCl, is possibly due to the reduced urinary kallikrein levels. In the animal hypertension experiments, it must be remembered that increase in the SBP on diet containing over 4% NaCl could be caused by reduced excretion of urinary active kallikrein.

### 4.2. Sodium Accumulation in Cells, Particularly in Blood Vessels

As mentioned above, in kininogen-deficient BN-Ka rats, which consumed a diet containing only 2% NaCl, NaCl is accumulated in the body and particularly in erythrocytes, accompanied by high blood pressure [145]. 

Even if high sodium is not consumed in the diet, sodium can be accumulated in erythrocytes and cerebrospinal fluid (CSF) by angiotensin II. In the normal BN-Ki rats, which take normal sodium diet (0.3% in diet), subcutaneous infusion of a non-pressor dose (20 μg/day/rat) of angiotensin II for two weeks with a mini-osmotic pump did not change the SBP, since it is a subpressor dose. However, the same treatment in kininogen-deficient BN-Ka rats, which take diet containing 0.3% NaCl, causes hypertension (180 ± 8 mmHg) [147], suggesting that hypertension may not be attributable to the direct vasoconstrictive action of angiotensin II. The heart rate and serum sodium levels were increased and the hematocrit values were reduced in the deficient BN-Ka rats. The sodium levels in erythrocytes rose gradually during subcutaneous infusion of angiotensin II in deficient BN-Ka rats and those in the CSF were also increased, suggesting that sodium is accumulated in the cells and CSF. These parameters were fairly constant in normal BN-Ki rats. Urinary active kallikrein and prokallikrein levels were slightly, but significantly, increased during the angiotensin II infusion in both strains. However, the elevated levels of urinary kallikrein in deficient BN-Ka were not different from those in normal BN-Ki rats. In contrast, urine volumes and urinary sodium excretion in deficient BN-Ka rats were actually not increased during angiotensin II infusion, whereas they were gradually increased in normal BN-Ki rats. Subcutaneous infusion of spironolactone, an aldosterone antagonist, was simultaneously carried out with angiotensin II in kininogen-deficient BN-Ka rats in the second week of the angiotensin infusion period. Spironolactone normalized, not only the high SBP, the heart rate, and urinary kinin levels, but also the sodium accumulation in erythrocytes and CSF to the levels in normal BN-Ki rats, indicating that the aldosterone, released by angiotensin II, induced both hypertension and the increase in sodium accumulation. Urinary secretion of aldosterone was increased during the angiotensin infusion, but there was no difference between the two strains of rats. Supplementation of LMW kininogen to kininogen-deficient BN-Ka rats during angiotensin II infusion normalized the SBP, heart rate, and urinary kinin levels and further, normalized the sodium levels in erythrocyte. In contrast, the administration of HOE 140 to normal BN-Ki rats increased these variables [147].

Sodium accumulation is observed not only in erythrocytes, but also in blood vessels. High sodium (8%) in diet increased high SBP of SD strain rats. Glibenclamide (a K_ATP_ channel blocker) reduced the increased SBP and increased the levels of urinary kallikrein and hence increased urinary sodium excretion [71]. Sodium contents in erythrocytes was increased by taking diet containing 8% NaCl. Glibenclamide (30 and 60 mg/kg) reduced the sodium levels in erythrocytes dose-dependently. A BK-B_2_ receptor antagonist, FR173657, reversed the deceased sodium levels of erythrocytes by glibenclamide. In the same experiments, the abdominal aorta was isolated and the connective tissues around the aorta were carefully removed. The NaCl contents in aorta took the same pattern as that of erythrocytes. Namely, increase of the sodium contents during taking 8% NaCl in diet was clearly decreased dose-dependently by glibenclamide (30 and 60 mg/kg), and the decreased sodium contents of the aorta were tended to be increased by additional BK-B_2_ antagonist, FR173657 [71]. 

### 4.3. Increased Sensitivity of Vascular Smooth Muscles to Angiotensin II and Norepinephrine after Sodium Accumulation

**Figure 6:**
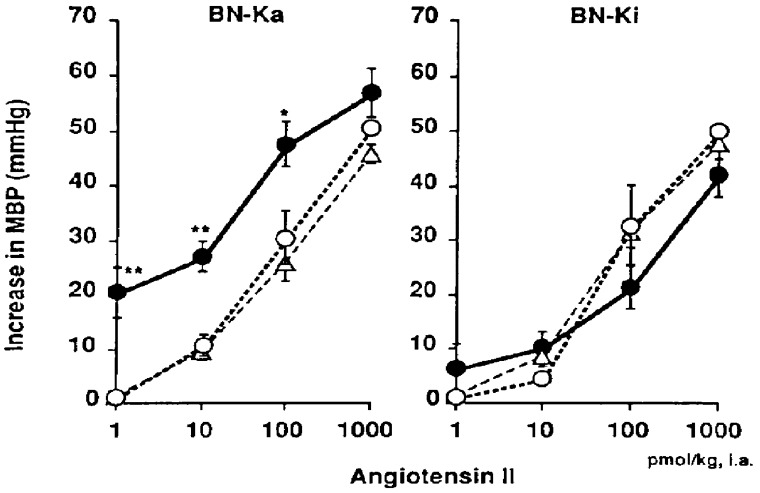
Changes in the elevation of the mean blood pressure (MBP) after a bolus intra-arterial injection of angiotensin II to conscious kininogen-deficient Brown Norway-Katholiek (BN-Ka) rats and conscious normal Brown Norway-Kitasato (BN-Ki) rats during infusion of two doses of NaCl. Values show the means ±S.E.M. from six rats. Sodium chloride solution (0.3 M or 0.15 M) was infused (6 mL/kg/hr) into the abdominal aorta for four days from 10 weeks of age. Values from rats infused with 0.3 M sodium chloride solution (closed circles) were compared with those infused with 0.15 M sodium chloride solution (open circles) at the same dose. *P < 0.05, **P < 0.01. Values represented by open triangles are those from untreated rats (quoted with permission from Ref. [137]).

Intra-arterial infusion of 0.3 M hypertonic NaCl solution into conscious, unrestrained kininogen- deficient BN-Ka rats caused significant increase in the mean arterial pressure. This infusion also caused increase in the sensitivity of the arterioles to vasoconstrictive substance [137]. As illustrated in Figure 6, the dose-response curves of the arterioles of kininogen-deficient BN-Ka rats for angiotensin II shifted to the left after infusion of 0.3 M NaCl, causing a approximately 10-fold increase in the arteriolar responses to angiotensin II, compared with the curve during 0.15 M NaCl (physiological saline). The arteriolar sensitivity to norepinephrine also increased by 20 times. In contrast, the sensitivity of the arterioles of normal BN-Ki rats did not change after infusion of either 0.15 or 0.3 M NaCl (Figure 6). The increased sensitivity in the arterioles of the deficient BN-Ka rat may be attributable to sodium accumulation in the vascular smooth muscle. These observations indicate that hypertension could be induced without increased plasma concentrations of any vasoconstrictive substances, which have been targeted by many researchers for the study of mechanisms of the development of hypertension. 

It was reported that cultured vascular smooth muscle cells from spontaneously hypertensive rats in the early stage show an enhanced Na^+^/H^+^ exchanger or an enhanced Na^+^ influx [148].

### 4.4. Sodium accumulation in cerebrospinal fluids and increased sympathetic discharge

Sodium accumulation in the cerebrospinal fluid is constantly observed in kininogen-deficient BN-Ka rats either taking a high sodium diet (2%) [145], upon intravenous infusion of 0.3 M NaCl [137], or by intravenous infusion of angiotensin II [147]. This is important for understanding the nature of hypertension, since this causes increase in sympathetic discharge. It is reported [149] that bolus injections of increasing concentrations of NaCl into the *cisterna magna* of SD strain rats enhances the discharge of the sympathetic nerves in the concentration-dependent manner and increases systolic blood pressure.

Increased sympathetic drive was observed frequently in young hypertensive patients, particularly during initial stage of hypertension [150]. A similar increase in responsiveness of the sympathetic nervous system was reported in hypertension models and hypertensive patients. 

## 5. Do Hypertensive Patients Secrete Less Urinary Kallikrein?

It is plausible that deficiency of BK-B_2_ receptors, tissue kallikrein, or kininogens develops hypertension by additional sodium intake, as above mentioned. On the other hand, is it true that hypertensive patients or hypertensive animal models secrete less kallikrein?

### 5.1. Hypertensive Patients

In 1934, significantly lower urinary kallikrein levels were reported [151] in hypertensive patients without clinically apparent renal disease, compared with those in normotensive subjects. Thirty-seven years later, Margolius *et al*. [59] described lower levels of urinary kallikrein in patients with essential hypertension than in a control population, and normal levels in patients with renal artery stenosis, and raised levels in patients with pheochromocytoma and primary aldosteronism. Subsequently, a large number of studies have been carried out in various human cases and animal models of hypertension. Patients with essential hypertension and low renin hypertension excrete lower urinary kallikrein than that of normal subjects [51,52,60,152,153,154,155,156,157,158,159,160]. However, this observation was blurred by a racial difference. Black hypertensive patients also excrete less urinary kallikrein than black normotensive subjects, but the mean values in normotensive blacks was not different from that in hypertensive whites during normal sodium intake [161]. As a result, the question of racial differences was raised.

During unrestricted sodium intake, urinary kallikrein activity was greater in white normotensives than in white hypertensives or black normotensives. On sodium restriction, all groups had greater urinary kallikrein activity than on unrestricted sodium intake, and the increase in black hypertensives was quite small [54]. 

An epidemiological survey also indicates that the urinary kallikrein concentration in random urine was significantly lower in black children than in white children and was positively correlated with urinary creatinine and urinary potassium concentrations, but is inversely related to the urinary sodium levels [53]. Families with the lowest kallikrein concentrations tended to have higher BP than did those with the higher concentrations, although the positive correlation was weak and was subject to many variables [53]. The significant inverse relationship between the urinary kallikrein levels divided by creatinine concentration and the BP in both white and black children was confirmed after 4 years of observation [162]. The familial aggregation of BP, BP rank, and concentration of kallikrein, in random urine were relatively stable in children over 8-year period of observation [162].

Segregation analysis of large numbers of Utah pedigrees, covering 1.2 million subjects as well as of 140,000 Utah death certificates over a 20-year period, was carried out to elucidate the genetic environmental determinants of hypertension, lipid abnormalities, and coronary arterial diseases [163]. According to the observations of these authors, kallikrein levels in approximately 30% of the population were low in “low homozygotes”, and such subjects have a high risk of hypertension. It is interesting that this percentage is close to that of 26% of the “salt-sensitive” population seen in the normotensive subjects [164]. Approximately 20% of this population consisted of “high homozygotes” of kallikrein, who had a low risk of hypertension whatever their potassium intake [163] (See the next section). On the basis of these observation, Williams *et al*. [163] proposed the following hypothesis: Approximately 50%, the rest of population, who were heterozygous for this single-gene trait, would be at high risk of hypertension if they followed a low-potassium diet, whereas a high potassium intake would reduce the risk of hypertension. This hypothesis is quite attractive, since suppression of hypertension with potassium intake has been repeatedly discussed. 

### 5.2. Salt-Sensitive Hypertension

It is widely believed that high sodium intake may induce hypertension in an entire population, as indicated by “INSALT” population study [165]. However, all population is not salt-sensitive. Definitions of the sodium sensitivity of BP have generally been based upon the difference between the BP after a low sodium intake, such as 9 mmol/day (ca. 0.5 g of NaCl), and that after a high sodium intake, such as 249 mmol/day (13.8 g) [166]. A difference of at least 10% between the mean arterial pressure after low-salt intake and that after high-salt intake is regarded as identifying salt-sensitivity, and a smaller difference as indicating a non-salt sensitive subject after salt loading [166]. Weinberger’s group used another stratagem for defining the salt sensitivity of the BP [164]: BP measurements were made after an intravenous infusion of three liters of normal (0.9%) saline for 4 h (308 mmol or 18 g of NaCl), and then on the next day, after sodium and volume depletion induced by a low sodium diet (10 nmol) and furosemide administration, in 378 healthy volunteers and 198 subjects with essential hypertension [164]. Those in whom mean arterial BP decreased by at least 10 mmHg after sodium and volume depletion were considered sodium-sensitive, and those with a decrease of 5 mmHg or less (including an increase in pressure) were considered sodium-resistant. It was found that 26% of the normotensive subjects were salt-sensitive and 58% salt-resistant, whereas in the hypertensive group, 51% were sensitive and 33% were resistant. It is worthy of mention that African-American are consistently more salt-sensitive than whites [167] and the sodium sensitivity of BP increases significantly with increasing age in all individuals [168]. Weinberger observed that 73% of black hypertensive patients were salt-sensitive, compared with 56% of a white hypertensive group [168]. 

There is no space here to discuss in depth salt-sensitive hypertension. Precise discussions on the interrelation of potassium, African-American, and salt-sensitive hypertension will be found in references [169,170]. However, from the observed results, it may not be surprising that black normotensive and hypertensive consume less potassium and tend to be more salt-sensitive, than their white counterparts. The key to these differences could be the renal kallikrein. 

A small-scale study was performed with 25 normotensive male and female subjects without any familial history of hypertension. They were divided into three groups on the basis of urinary kallikrein excretion (low, normal, and high) [171]. After sodium loading, the urinary excretion of active kallikrein decreased in all three groups to the same degree while sodium excretion increased. The systolic BP increased significantly in the low-kallikrein group, but remained unchanged in the normal kallikrein group, and showed a tendency to decrease in the high-kallikrein group (low +4.6 ± 1.6. *p* < 0.01; normal +1.2 ± 2.8; high -2.1 ± 2.1 mmHg: low vs high: p < 0.0025). Considering all groups together, there was a significant inverse linear relationship between the change in BP during sodium loading and the urinary kallikrein excretion at maximum sodium restriction (systolic BP r = -0.4354, *p* < 0.05).

With a relatively small group of 37 hypertensive male patients, a randomized crossover, double-blind study was performed [172]. The patients were classified as salt-sensitive, when diastolic BP changed 10 mmHg or more occurred after NaCl intake in both low NaCl period (40 mmol per day for two weeks) and the high NaCl period (240 mmol NaCl per day for two weeks). Nineteen hypertensive patients were salt-sensitive, while 18 were classified as salt-resistant. The urinary excretion of active kallikrein was significantly lower (p < 0.0001) in salt-sensitive patients (0.51 ± 0.36 U/24 h) than in salt-resistant patients (1.28 ± 0.48 U/24 H). These clinical studies were carried out with only a small number of patients, but if large scale of clinical studies will be performed to search the relationship between salt-sensitivity and urinary kallikrein levels, urinary kallikrein might become a good parameter to classify the salt-sensitive group. A large scale of clinical studies to clarify the reduced levels of urinary kallikrein as a marker of the salt-hypertension awaits future studies [170]. 

### 5.3. Hypertensive Animal Models

#### 5.3.1. DOCA-Salt Hypertension Model

DOCA-salt-induced hypertension is a commonly used hypertension model in rats. Rats underwent unilateral nephrectomy at seven weeks of age and after the nephrectomy, rats were injected by deoxycorticosterone acetate (DOCA, 5 mg/kg daily) and given by 1% sodium in drinking water [132]. The SBP of normal BN-Ki rats increased gradually and reached a plateau at 11 to 12 weeks after the start of treatment (18 to 19 weeks of age). In contrast, kininogen-deficient BN-Ka rats rapidly raised the SBP after the start of the treatment and reached a plateau at five weeks. This indicates that the renal kallikrein-kinin system plays a suppressive role in the developmental stage of hypertension in normal rats. Urinary kallikrein and prokallikrein started to increase immediately after the onset of the treatment in both normal BN-Ki and kininogen-deficient BN-Ka rats, reaching peaks at 10 weeks of age and declining thereafter [173]. There was no difference in urinary kallikrein excretion between the two strains. However, the urine volume and urinary sodium increased only in normal BN-Ki rats, the levels in deficient BN-Ka rats remaining unchanged because of the lack of kininogen and kinin generation. It is important to point out that when the urinary kallikrein secretion in normal BN-Ki rats peaked off at 10 weeks of age, a marked increase in the SBP was observed. Uninephresctomy + DOCA injection without supplement of 1% NaCl in drinking water in Sprague-Dawley rats did not raise the SBP, indicating that supplement of salt is important [173] (see also Figure 8). The anti-hypertensive effect of kidney-specific kininase inhibitors in this model will be discussed later. 

#### 5.3.2. Spontaneously Hypertensive Rats (SHR)

Okamoto-Aoki’s SHR strain was separated from Wistar Kyoto (WKY) strain [174]. In this SHR strain, urinary excretion of kallikrein was subnormal [175,176,177]. A time-course study [176] revealed that the urinary excretion of active and total kallikrein was significantly lower in SHR on a normal sodium diet from four through 15 weeks of age. The average values of active and total kallikrein activity in these SHR were 69.5% and 67.4%, respectively, of those values in age-matched WKY at all stages of the development of hypertension and even after the SBP reached a plateau at 10 to 11 weeks. SHR exhibit a lower urinary excretion of sodium and water than did WKY rats together with a higher cumulative sodium balance at all ages studied and a higher cumulative water balance at seven and eight weeks of age [178]. These strain differences were related neither to urine flow nor sodium excretion nor to glomerular filtration rate [176].

The reduced urinary kallikrein excretion in SHR was observed particularly at the developmental stage of hypertension. The levels of urinary kallikrein in SHR were significantly lower than those in WKY rats. The difference was largest in weanlings (four weeks of age), but the difference in the urinary kallikrein level between SHR and WKY disappeared when the SBP has reached a plateau at the age of 10 weeks [179]. This result disagrees with the report that a lowered excretion of urinary kallikrein persists after the blood pressure has reached a plateau [178]. Renin activity was higher in SHR weanlings (four weeks of age) than in WKY weanlings, indicating that SHR may be prone to accumulate sodium through aldosterone release in the neonatal stage. Reduced daily excretions of not only sodium, but also potassium and creatinine were observed in SHR weanlings from the age of four weeks, in comparison with corresponding levels in WKY, although the serum level and the urinary excretion of creatinine in both strains were within the normal range [179]. In contrast, it was reported that, in studies on clearance and micropuncture study of the kidney, abnormalities in glomerular function were observed during the development of hypertension in 6-week-old SHR [180]. It was also reported that SHR of NIH F22-24 strain excrete less kallikrein at 23 weeks, and that the level was not increased by dietary sodium restriction [181]. In studies on the renal parenchymal values and immunoreactivity of tissue kallikrein in Okamoto SHR aged to four to 78 weeks [182], the enzymatic activity of renal tissue kallikrein (active and total) increased from four weeks in SHR compared with normotensive WKY in association with a significant increase in the blood pressure of SHR. In view of the reduced urinary excretion of kallikrein, the greater values for renal kallikrein in the kidney tissue during the early phase of SHR may be explained by a primary defect in the mechanisms that regulate release of renal kallikrein from CNT cells. In contrast, the renal tissues of 78-week-old SHR and human renal biopsy tissues showed a substantial reduction in tissue kallikrein values. Both renal tissues showed a reduction in immunoreactivity in the cells of the connecting tubules, probably secondary to a loss of distal tubular mass, as a result of tubular atrophy and fibrosis [182].

In dissected renal connecting tubules of young SHR and WKY rats (4-6 weeks old), the secreted levels of kallikrein in SHR was not different from those of WKY rats before stimulation, but the significant increase was observed after placing potassium on the tubules at the concentration of 20-70 mM in WKY rats [69]. In contrast, potassium induced negligible increase in secretion of kallikrein in SHR. Kidney-specific K_ATP_ channel blocker, PNU-37883A (0.1, 1. 10, and 100 μM) also accelerated significant increase of secretion of renal kallikrein in WKY rats, but, in SHR, PNU37883A secreted the kallikrein only slightly. This indicates that in SHR, some difficulty may be present in secretory process of renal kallikrein [69].

#### 5.3.3. Dahl Salt-Sensitive Rats

Hypertension induced by ingestion of excess sodium in Dahl salt-sensitive rats is caused by an interaction of both genetic and environmental factors. L-3,5,3’-triiodothyronine with 7.3% NaCl to SD strain rats resulted in two opposite predispositions to hypertension caused by NaCl ingestion in offspring; one group became salt sensitive and hypertension prone (S), and the other salt resistant and hypertension resistant (R) [183]. It was shown that when R strain rats were united to S strain rats in parabiosis, the former developed sustained hypertension when a high-NaCl diet was consumed by the pair [184,185,186]. Dahl S rats also developed hypertension both after injection of DOCA-salt or unilateral renal artery compression without salt [183,187] and after cortisone administration or adrenal regeneration [188]. In DOCA-salt hypertension, Dahl S rats became hypertensive more rapidly than did R rats [187]. This finding suggests that S rats might share the feature of kininogen-deficient BN-Ka rats, which increased SBP rapidly and easily accumulated sodium in the body through the failure of kinin generation by the kidney (See Section 3.3.).

Another study on the transplantation of kidneys to bilaterally nephrectomized recipients revealed that the blood pressure of the cross-transplanted groups was intermediate between those of the control groups with transplanted kidney (R/R and S/S), where the kidney genotype/recipient genotype is indicated by R/S and S/R. The rank order of urinary kallikrein excretion was R/R = R/S > S/R = S/S [189].

Dhal S rats had less urinary kallikrein activity that did R rats [190,191]. A decrease in urinary kallikrein activity may induce salt sensitivity, as in other animal models. However, this difference is not simply interpreted as a reduced excretion of renal kallikrein, because the urinary protein excretion rate in S rats became greatly elevated (proteinuria) as the hypertension developed [192,193]. The daily administration of dexamethasone for seven days caused marked suppression of urinary kallikrein excretion in both S and R rats, together with increase in urinary protein in S rats, but not in R rats [194]. Treatment with DOC increased urinary kallikrein in R rats but not S rats, while S rats responded to sodium deficiency with increased urinary kallikrein excretion. Mild glomerular and distal tubular scarring was found in S rats, and these lesions are quite comparable with increases in blood pressure and proteinuria. No such lesions appeared in the controls or the DOCA-treated R-rats [195]. 

In Dahl S rats fed normal sodium diet (0.45% NaCl), the urinary kallikrein levels assessed on the basis of the kinin-generating activity was lower than the level determined by direct radioimmunoassay of the enzymatic protein. The lower level of kallikrein may have been due to the action of inhibitors leaking from the plasma [161]. This interpretation is opposed by another report [190] that the lower level of urinary kallikrein is due to decreased excretion of renal kallikrein rather than to greater amounts of inhibitors in the urine, and to abnormality in the enzyme, or an inactive enzyme. Furthermore, another report [196] stated that the isoelectric focusing patterns of the urinary kallikrein of S rats showed that their kallikrein has a lower sialic acid content than that of R rats, and that treatment of kallikrein from R rats with neuraminidase converted it to the S-type pattern on the gel.

#### 5.3.4. Other Genetically and Experimentally Hypertensive Rats

Genetically hypertensive New Zealand strain rats excreted reduced levels of urinary kallikrein [197]. The urinary excretion of kallikrein by hypertensive fawn-hooded (FH/Wjd) male and female rats was less than that of Wister rats (and males excreted less than female) from 1.5 months before the hypertension developed at the ages of two months (males) and 4.5 months (female) [198]. FH male rats excreted more sodium and urine than did any other groups. Only FH male rats developed proteinuria, but neither an inhibitor or urinary kallikrein nor increased degradation of this enzyme in the urine was found [198]. Milan hypertensive strain (MHS) rats [199] also secreted less urinary kallikrein [200]. 

Rats with renovascular hypertension have decreased kallikrein levels both in renal tissue and in urine [152]. In two-kidney, one-clip Goldblatt hypertensive rats, the urinary kallikrein levels were low in the urine of the stenotic kidney but normal in that of the contra-lateral kidney [200].

In other hypertensive animals, the urinary kallikrein activity may be reduced. It is possible that the reduction is attributable to concomitant impairment of renal function and, further, the renal impairment may be secondary to continuing hypertension. Thus, the reduced excretion of urinary kallikrein should be carefully distinguished from the reduction of urinary kallikrein activity due to renal inpairement. 

## 6. Antihypertensive Effects of Renal Kallikrein Releasers and of Urinary Kininase Inhibitors on Salt-Induced Hypertensive Models

### 6.1. Agents, Which Accelerate Release of Renal Kallikrein

Many studies suggest that potassium intake is inversely related to systolic and diastolic blood pressure. Potassium deficiency may play a special role in the high incidence and prevalence of hypertension in blacks [201]. The reduction of the systolic and diastolic blood pressure by increased potassium intake is more pronounced in hypertensive patients, compared to normotensive persons, in blacks compared to whites, and in those consuming a high intake of sodium [201]. From the above-mentioned evidences (Section 2.5.3.), we firmly believe that potassium administration directly secretes renal kallikrein.

**Figure 7:**
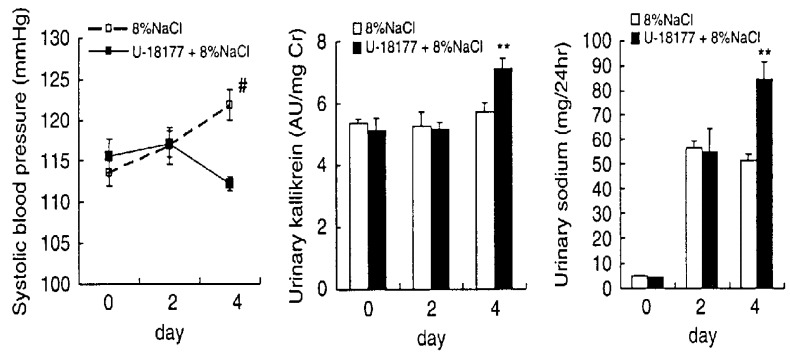
Effects of a kidney-selective ATP-sensitive potassium channel blocker (U-18177) on systolic blood pressure (left), urinary kallikrein levels (middle) and urinary sodium levels (right) in Sprague-Dawley strain SD rats fed on an 8% NaCl diet. U18177 (30 mg/kg twice a day) was given to rats on 8% NaCl diet (closed squares and columns). The values were compared with the values of rats given vehicle and 8% NaCl in their diet (open squares and columns). *P < 0.05, ** P < 0.01 *vs*. 8% NaCl in the diet on day 4 (reproduced from Ref. [71]).

As one of anti-hypertensive agents, ATP-sensitive potassium (K_ATP_) channel blockers, particularly, the kidney-specific K_ATP_ channel blocker, are one of the most promising anti-hypertensive agents for salt-sensitive hypertensive patients. In fact, as shown in Figure 7, the increased SBP (122 ±2 mmHg) of vehicle-treated SD strain rats, induced by 8% NaCl in diet on day 4, was greatly suppressed by administration of a kidney-specific K_ATP_ channel blocker (U18177, 30 mg/kg/twice a day, p.o.) to 112 ± 1 mmHg (P < 0.05). The reduction of SBP by U18177 was accompanied with significant increase in urinary kallikrein levels on day 4 (7.1 ± 0.3 AU/mgCr), compared with the level in vehicle- treated rats on day 4 (5.8 ± 0.2 AU/mgCr)(P < 0.01). Consequently, urinary sodium levels were largely increased to 84.5 ± 7.3mg/24 hr in U18177-treated rats, compared with 50.5 ± 2.1 mg/24 h in vehicle treated rats (P < 0.01) [71]. 

Hypertensive patients, particularly, salt-sensitive hypertensive patients, are thought to excrete less urinary kallikrein. Supplementation of potassium has already widely recommended for hypertensive patients. K_ATP_ channel blockers will be added as a new type of the antihypertensive drugs. The mechanism of actions of K_APT_ channel blockers was precisely discussed in Section 2.5.4. Glibenclamide cannot be used to hypertensive patients, as far as patients are not affected by diabetes mellitus, because of avoiding hypoglycemia. Kidney-selective K_ATP_ channel blocker, PNU-37883A, has already been developed. Unfortunately this agent has been discarded from the market, since its diuretic action is week under normal salt intake. If this agent would be tested under high sodium intake, the future course of the agent may have been changed. PNU-37883A is 1,000 times more potent than glibenclamide in kidney slice experiments, as above mentioned. As the intravenous administration of PNU37883A required the similar dose to that of glibenclamide [66], further structural modification of the agent may be required for improving better stability in vivo. If the reduced levels of renal kallikrein in urine are a major mechanism of development of salt-sensitive hypertension, this type of the agent will be one of the most reliable antihypertensive drugs.

### 6.2. Inhibitors of Renal Kininase (Kinin-Degradation Enzyme)

As precisely discussed in Section 2.4., urinary kinin-destroying enzymes (kininases) are completely different from those in plasma, so that ACE inhibitors are ineffective for degradation of urinary kinin, which is released by renal kallikrein in the cortical CD. Urinary kinins in the cortical collecting tubules of the kidney are destroyed by carboxypeptidase-Y-like exopeptidase (CPY) and neutral endopeptidase (NEP). Ebelactone-B inhibits mainly CPY, a major kininase in rat and human urine. Poststatin inhibits both CPY and NEP. Administration of either ebelactone B or poststatin to rats increased urinary kinin level and urine volume and increase excretion of sodium and chloride.

**Figure 8:**
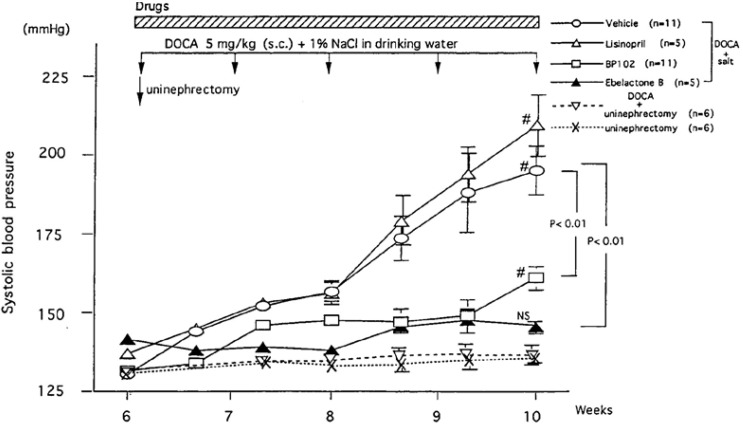
Effects of ebelactone B, BP102, and lisinopril on the development of deoxycorticosterone acetate (DOCA)-salt hypertension. Values are means ±S.E.M. After unilateral nephrectomy at six weeks of age, deoxycorticosterone acetate (5 mg/kg, s.c.) was administered once a week, and ebelactone B (5 mg/kg), BP102 (30 mg/kg), or lisinopril (5 mg/kg) was administered orally twice a day from immediately after the surgery for 4 weeks. Values in rats receiving these three compounds are compared with those in rats receiving vehicle (open circle) at 10 weeks of age. The results of the unilaterally nephrectomized group with DOCA and the unilaterally nephrectomized group without DOCA-salt are shown as the control. # P < 0.05, the values at 10 weeks of age were compared with those at 6 weeks. ANOVA was used to evaluate the significance of difference (reproduced from Ref. [202], with permission).

Figure 8 depicts the anti-hypertensive effect of inhibitors of urinary CPY and NEP in the DOCA-salt hypertension model of SD-strain rats [202]. DOCA-salt hypertension was induced both by weekly subcutaneous injection of deoxycorticosterone acetate (DOCA) solution (5 mg/kg/week) and by 1% NaCl in drinking water in uni-nephrectomized SD rats of six weeks of age. The SBP of DOCA-treated rats gradually increased to 155 ±3 mmHg and 195 ± 7 mmHg (eight and 10 weeks of age, respectively), while the SBP of non-treated rats was 137 ± 2 mmHg and was not changed. The kininase inhibitors were orally administered twice a day for four weeks from the first day of DOCA-salt treatment. Lisinopril, an ACE inhibitor, did not suppress the development of hypertension. Ebelactone B (5 mg/kg), a urinary CPY kininase inhibitor, almost completely suppressed the development of hypertension. For NEP, an another renal kininase, BP102 (sinorphan, [(*S*)-2-[(acetylthio)methyl]-1-oxo-3-pheylpropyl]glycine benzyl ester) [203,204], which was developed as an oral prodrug of NEP inhibitor, thiorphan, largely suppressed the development of hypertension. SBP of two control groups, uni-nephrectomized rats and uni-nephrectomized + DOCA rats, was not changed. The suppressed SBP in the DOCA-sat hypertensive rats by ebelactone B was reversed by continuous subcutaneous infusion of HOE 140, a BK-B_2_ receptor antagonist, to 167 ± 3 mmHg. Again, the suppression of the SBP by ebelactone B was attributed to increase in urinary kinin levels or to enhanced activity of the renal KKS. 

In normal BN-Ki rats of eight weeks of age, the SBP in DOCA-salt hypertension was suppressed by ebelactone B (5 or 15 mg/kg/day), while, as expected, in kininogen-deficient BN-Ka rats of the same age, the SBP was not suppressed, since urinary kinin was absent [41].

These results clearly indicate that inhibitors of urinary kininases, CPY and NEP, are effective in hypertension models.

## 7. Roles of KKS on Cardiovascular Disorders and Angiogenesis and a Therapeutic Aspect of Tissue Kallikrein Gene Delivery

The present review concentrates on the role of the renal KKS, particularly against development of salt-sensitive hypertension. However, recent studies on the protective roles of bradykinin or KKS in cardiovascular diseases have been largely extended, so that roles of KKS in cardiovascular diseases and therapeutic approaches by kallikrein gene delivery will be briefly reviewed in this section. 

### 7.1. Cardiac Ischemia and ACE Inhibitors

It was earlier reported that inhibitors of ACE, which is identical to kininase II, protect against cardiac ischemia in *in vivo* and *ex vivo* models by limiting infarct size [205] and protect against reinfarction [206]. Schölkens *et al*. reported that bradykinin antagonism by HOE 140 largely abolished these protective effects of ACE inhibitor, ramipril, in isolated ischemic heart of guinea pigs or rats [207]. The beneficial effects of ACE inhibitors are reviewed in detail in the references [208,209]. The protective effect of ramipril was abolished by HOE 140, nitric oxide synthase inhibitor (NG-nitro-L-arginine methyl ester) or indomethacin [210]. Administration of ACE inhibitors, through increased kinin levels, together with NO formation and prostaglandin (PG) I_2_ release in the endothelial cells, causes blood pressure reduction in high renin (two-kidney, one-clip) renovascular model of hypertension, and in the balloon denudation model of the carotid arteries in rats, ACE inhibitors caused neointimal formation or preservation of the endothelial function and vascular reactivity, and further, in cardiac ischemic-reperfusion model, the incidence and the duration of ventricular fibrillation and the improvement of cardiodynamics and myocardial metabolism were reduced by ACE inhibitors [208]. Ramipril, a ACE inhibitor, also prevents left ventricular hypertrophy and fibrosis [211]. Aortic constriction induced the pressure-overloaded cardiac hypertrophy in mice after 14 to 28 days. BK-B_2_ receptor mRNA in the hypertrophic hearts rapidly decreased in the heart within seven days and BK-B_2_ receptor densities in cardiac membrane preparations significantly decreased four days after the aortic constriction, but the receptor affinity was unchanged. Down-regulation of BK-B_2_ receptor mRNA levels was abolished by oral administration of an angiotensin II type I agonist, candesartan [212]. Rats harboring the human tissue kallikrein gene significantly reduced overflow of nucleotide breakdown products upon reperfusion after cardiac ischemia [213]. Bradykinin perfusion of isolated working hearts reduced the incidence of post-ischemic reperfusion arrhythmias as well as duration of ventricular fibrillation, improvement of cardiodynamics via increased left ventricular pressure, contractility, and coronary flow without changes in heart rates [214]. In rats, when the coronary artery of rats was permanently ligated, the myocardial infarct size of kininogen-deficient BN-Ka rats is 1.3-fold larger than that in normal BN-Ki rats at 12, 24, and 48 h after ligation. The regional myocardial blood flow around the necrotic lesion in deficient-BN-Ka rats, measuring by microsphares, was reduced by 41–46%, compared with that of normal BN-Ki rats. Infusion of a nonpeptide B_2_ receptor agonist, FR190997, into vena cava of BN-Ka rats for 24 hours caused significant reduction in the myocardial infarct size [215]. 

### 7.2. Ischemic Preconditioning and Reperfusion of the Heart

BK and related kinins also seem to be involved in preconditioning and remodeling. It was first reported in 1986 [216] that brief episodes of ischemia/reperfusion protect the myocardium from the infarct size induced by subsequent more prolonged ischemia. Since then, preconditioning has been known for protecting the heart against ischemia/reperfusion injury in various species of animals, such as dogs, pigs, rabbits, mice and rats [217,218]. Kinin-generating pathways are present in the heart and kinins are released under conditions of ischemia [219]. The ventricular myocardium of adult male rats expressed mRNA for tissue kallikrein, BK-B_2_ receptors, and low molecular weight kininogen, and T-kininogenase and T-kininogen, but not those for high molecular weight kininogen and BK-B_1_ receptors [220]. Bradykinin and transient ischemia-mediated preconditioning was eliminated by co-infusion of the B_2_-receptor antagonist [221]. Short preconditioning of myocardial ischemia protected the myocardium against arrhythmogenic effects of a more prolonged occlusion, and this protection is lost by cyclooxygenase inhibitor, meclofenamate [222]. Bradykinin infusion in isolated rabbit hearts mimicked preconditioning and protection and was not affected by pretreatment with the nitric oxide synthase inhibitor, *N*-ω-nitro-L–arginine methyl ester, or the prostaglandin synthase inhibitor, indomethacin, but could be completely abolished by the protein kinase C inhibitors, polymyxin B and staurosporine as well as by a BK-B_2_ receptor antagonist, HOE 140 [223]. In BK-B_2_ receptor knockout mice, the protective effect by preconditioning is absent [224]. The increase in the interstitial BK concentration occurs earlier than the increase in the interstitial adenosine concentration in pigs [225], and its interstitial release during the index ischemia is enhanced by ischemic preconditioning [226]. Bradykinin is a necessary trigger only during a shorter duration of preconditioning ischemia in pigs [225], or single cycle of ischemia/reperfusion in rabbits [223], but during a more prolonged period (10 min) of preconditioning ischemia [225] or repeated cycles of ischemia/reperfusion [223], the interstitial adenosine concentrations were increased and adenosine is more important. In pigs, BK-B_2_ receptor blockade by HOE 140, combined with increased breakdown of endogenous adenosine by adenosine deaminase completely abolishes the infarct size reduction achieved by an prolonged period (10 min) of preconditioning stimulus [225]. 

Ischemic preconditioning significantly reduces infarct size by 65% in wild-type mice (WT) and by 40% in tissue kallikrein deficient mice (TK-/-) (P < 0.05, TK-/- vs WT) [227]. However, this report on the effective role of tissue kallikrein in ischemic preconditioning is controversial to the results that rats deficient in only high-molecular weight kininogen did not prevent the ratio of infarct size to risk area [224], since high molecular kininogen in plasma is consumed only by plasma kallikrein, not by tissue kallikrein. Further study will be required. 

In human studies, repetitive balloon inflations or intermittent aortic clamping during angioplasty or coronary bypass surgery provides similar cardioprotection in patients [228,229]. Intracoronary infusion of bradykinin before percutaneous transluminal coronary angioplasty (PTCA) rendered the myocardium relatively resistant to subsequent ischemia [230]. As a wide variety of studies were carried out, many reviews have been published [208,231,232,233,234,235,236]. 

### 7.3. Angiogenesis

Tissue kallikrein knockout mice (KLK-/-) show impaired muscle neovascularization in response to hindlimb ischemia. Injection of adenovirus-mediated human tissue kallikrein (hKLK1) was able to rescue this defect. Similarly, in the rat mesenteric assay, adenovirus-mediated hKLK1 induced a mature neovasculature with increased vessel diameter through BK-B_2_ receptor mediated recruitment [237]. 

Marked neointima formation occurs after balloon injury in rat arteries. Angiotensin II has been implicated as a growth factor and kinin is known to stimulate NO and PGI_2, _both of which has antigrowth effects [238]. Both ramipril (an ACE inhibitor) and losartan (an AT_1_-type angiotensin II receptor antagonist) significantly reduced neointima formation and ramipril had a more marked effect [238]. The kinin antagonist HOE 140 reduced the inhibitory effect of ramipril by 73% and NO synthesis inhibitor, L-NAME, also reduced the effect of ramipril and losartan [238]. Kallikrein gene transfer into cultured rat vascular smooth muscle cells resulted in time-dependent secretion of recombinant human tissue kallikrein and inhibition of cell proliferation [239]. Balloon angioplasty reduced endogenous rat tissue kallikrein mRNA and protein levels at the injured site. In rats, which receive adenovirus-mediated human tissue kallikrein gene delivery, a 39% reduction in intima/media ratio at the injured vessel after delivery compared with that of rats received control virus. HOE140 blocked the protective effect and reversed the intima/media ratio to that of the control rats [239]. The intima hyperplasia and media thickening after the left carotid artery ligation were partially suppressed by captopril (an ACE inhibitor), but this suppression was partially reduced by a BK-B_1_ antagonist or a BK-B_2_ antagonist, or in B_2_ receptor deficient mice [240]. 

BK-B_2_ receptors were abundantly present on CD133(+) and CD34(+) circulating angiogenic progenitor cells (CPCs) as well as cultured endothelial progenitor cells (EPCs) derived from blood mononuclear cells [241]. In transwell migration assays, BK exerts a potent chemoattractant activity on CD133(+) and CD34(+) CPCs and EPCs via BK-B_2_ receptor/phosphoinositide 3 kinase/eNOS-mediated mechanism. Migration toward BK was able to attract an blood mononuclear cell subpopulation enriched in CPCs with *in vitro* proangiogenic activity, as assessed by Matrigel assay [241]. Reduction of BK sensitivity in progenitor cells from cardiovascular disease patients might contribute to impaired neovascularization after ischemic complication [241]. Probably, further studies are necessary on the role of BK in neointima formation.

Recent evidences suggest that angiogenesis is an important mechanism in tumor development and was predominantly observed in the stroma microenvironment. Angiogenesis induced by sponge implants in rats was significantly suppressed in kininogen-deficient Brown Norway Katholiek (BN-Ka) rats, compared with that in normal Brown Norway Kitasato (BN-Ki) rats [242]. The angiogenesis enhanced by basic fibroblast growth factor was also significantly less marked in deficient BN-Ka rats than in normal BN-Ki rats. Angiogenesis in normal BN-Ki rats was significantly suppressed by BK-B_1_ or -B_2_ receptor antagonist. mRNA of vascular endothelial growth factor (VEGF) was more intensely expressed in the granuloma tissues of normal BN-Ki rats than in deficient BN-Ka rats. Daily topical injection of aprotinin, a kallikrein inhibitor, suppressed angiogenesis. Daily injections of low-molecular weight kininogen enhanced angiogenesis in deficient BN-Ka rats. FR-190997, a nonpeptide mimic of bradykinin, promoted angiogenesis markedly with concomitant increase in vascular endothelial growth factor mRNA [242]. In mice bearing sarcoma 180 cells, a BK-B_2_ receptor antagonist, HOE 140, significantly suppressed the increment in angiogenesis and tumor growth, but a BK-B_1_ receptor antagonist, desArg10-HOE 140 did not [243]. The difference in suppressive effects of BK_1_ or BK_2_ receptor antagonists might be dependent to types of the tumor. In mice bearing sarcoma 180 cells, the vascular permeability was significantly enhanced in the early phase (peaked at day five), whereas tumor angiogenesis increased gradually over 20 days [244]. BK-B_2_ receptor antagonist, FR173657, significantly suppressed the vascular permeability, but a B_1_ agonist, desArg10-HOE 140 did not. Immunoreactive B_2_ receptor was present in the endothelial cells in the early phase, whereas B_2_ receptors were also observed in the stromal fibroblasts in the late phase. VEGF immunoreactivity was detected exclusively in the stromal fibroblasts only in the late phase. VEGF immunoreactivity was attenuated by FR173657, a B_2_ receptor antagonist. Tumor angiogenesis was significantly reduced by treating the tumor tissues with FR173657 both in the early phase and in the late phase [244]. 

Subcutaneous inoculation of Walker 256 carcinoma cells into normal BN-Ki rats resulted in the rapid development of solid tumors with marked angiogenesis, whereas, in the kininogen-deficient BN-Ka rats, the tumor weight and extent of angiogenesis were significantly less than those in normal BN-Ki rats [245]. Angiogenesis and tumor growth were significantly suppressed in BK-B_2_ receptor knockout mice bearing sarcoma 180 compared with their wild-type counterparts. Immunoreactive VEGF was localized in Walker tumor stroma more extensively in normal BN-Ki rats than in deficient BN-Ka rats. Immunoreactive BK-B_2_ receptor was detected in the stroma to the same extent in both strains of rats. Cultured stroma fibroblasts isolated from BN-Ki and BN-Ka rats produced VEGF in response to BK [245]. 

High molecular weight kininogen (HK) plays important roles in fibrinolysis, thrombosis, and inflammation. HK binds to endothelial cells where it can be cleaved by plasma kallikrein to release BK. The remaining portion of the molecule, cleaved HK, is designated cleaved high molecular weight kininogen or HKa[246]. HKa inhibits tube forming capacity of endothelial progenitor cells by suppression of matrix metalloproteinase-2 activation [247].

Tissue kallikrein protects against acute phase myocardial infarction by promoting neovascularization, restoring regional blood flow and improving cardiac function through the kinin B_2_ receptor-Akt-glycogen synthase kinase-3beta and VEGF signaling pathways [248]. As a wide variety of studies on angiogenesis were carried out, many reviews have been published [246,249,250,251,252,253,254].

### 7.4. Tissue Kallikrein-Gene Delivery

As an alternative approach to control hypertension or other cardiovascular diseases, human tissue kallikrein gene therapy has been proposed [255,256]. The kallikrein gene was delivered into spontaneously hypertensive rats (SHR) via intramuscular, intravenous, portal vein, intraperitoneal, and intra-cerebroventricular routes. A single injection of naked human kallikrein DNA constructs caused a prolonged reduction of high blood pressure for up to eight weeks. Adenovirus-mediated human tissue kallikrein gene delivery also results in high efficacy of human kallikrein expression. Immunoreactive human kallikrein was detected in rat serum at the highest level at one day post gene delivery [255]. The hypotensive effect caused by somatic gene delivery of human tissue kallikrein in hypertensive rats is reversed by aprotinin, a potent tissue kallikrein inhibitor [256] or HOE 140, a specific BK-B_2_ receptor antagonist [257,258]. The expression of human tissue kallikrein in rats was identified in the heart, kidney, aorta, lung and liver by reverse transcription-polymerase chain reaction followed by Southern blot analysis and by ELISA [256,259]. Adenovirus-mediated kallikrein gene delivery caused delay in the increase in blood pressure from day 2 to day 41 post injection in SHR, but no effect on the blood pressure of normotensive WKY rats. Immuno-reactive human tissue kallikrein can be detected in sera and urine of rats receiving kallikrein gene delivery. Urinary kinin and cyclic GMP (cGMP) levels were significantly increased in rats receiving kallikrein gene delivery [260]. Adenovirus-mediated human kallikrein gene delivery in Dahl salt-sensitive rats induced prolonged reduction of blood pressure and increased urinary kinin and cGMP levels, inhibition of cardiac hypertrophy, and attenuation of renal injury [261,262]. A single intravenous injection of adenovirus carrying the human tissue kallikrein gene into two-kidney, one-clip Goldblatt hypertensive rats caused a significant reduction in the left ventricular mass and cardiomyocyte size, as well as increase in renal blood blow, urine flow, glomerular filtration rats, electrolyte output, and urine excretion, together with significant increase in urinary kinin, nitrite/nitrate, and cGMP levels [263]. Adenovirus-mediated kallikrein gene delivery in DOCA-salt rats caused a significant reduction in blood pressure, urinary secretion, urinary protein levels and body weight. Morphological examination of the kidney showed that the gene transfer significantly reduced glomerular sclerotic lesions, brush border disruption of proximal tubules, tubular dilatation and protein cast accumulation [264]. Kallikrein gene transfer in the same model significantly decreased proteinuria, glomerular sclerosis, tubular dilatation, and luminal protein casts, levels of collagen I and fibronectin [265]. The kallikrein gene transfer also reduced kidney weight, glomerular size, proliferating tubular epithelial cells and macrophages/monocytes and TGF β_1_ immunostaining in glomeruli. Furthermore, in chronic renal failure of rats with 5/6 reduction of renal mass, human tissue kallikrein gene delivery attenuates hypertension, renal injury, and cardiac remodeling [266]. It is possible that delivery of human tissue kallikrein gene may compensate the deficiency of the renal kallikrein secretion from the CNT cells of the kidney in hypertensive models.

Tissue kallikrein reverses insulin resistance and attenuates nephropathy in diabetic rats [267]. Intramuscular delivery of adenovirus-mediated human tissue kallikrein gene in mice induced angiogenesis in the muscle and increased muscular blood flow [268]. In streptozotocin-induced diabetic rats, local delivery of human tissue kallikrein gene halted the progression of microvascular rarefaction in hind limb skeletal muscle by inhibiting apoptosis, thus ensuring an improved hemodynamic recovery in case of supervening vascular occlusion [269]. 

Kallistatin (a tissue kallikrein-binding protein) is a potent vasodilator, which may function directly through a vascular smooth muscle mechanism independent of an endothelial bradykinin receptor [270]. It is also reported [271] that kallistatin induces vasorelaxation of isolated aortic rings and reduced renal perfusion pressure in isolated rat kidneys. Transgenic mice overexpressing rat kallistatin are hypotensive, and adenovirus-mediated gene delivery of human kallistatin attenuates blood pressure rise in SHR [271]. Kallistatin stimulates the proliferation and migration of vascular smooth muscle cells *in vitro* and neointimal formation in balloon-injured rat arteries. Kallistatin inhibits the proliferation, migration and adhesion of endothelial cells *in vitro* and angiogenesis in the rat model of hind limb ischemia [271]. 

## 8. Proposal of a New Category of Anti-Hypertensive Drugs against the Salt-Sensitive Hypertension

Human kallikrein gene delivery therapy may be one of the hopeful candidates for control of hypertension, but it may require careful techniques and an ethical decision. Furthermore, it was reported [272] that in transgenic mice, expression of human tissue kallikrein appears to exert a profound effect on the cytoarchitecture of lymphatic tissues and a general decrease in lymphocytes, particularly in T cell-dependent areas. These findings presumably reflect altered function of lymphatic tissue in transgenic mouse strains carrying the human kallikrein gene. Thus, further studies may be necessary for clinical use of the tissue kallikrein gene therapy.

Oral administration of drugs is an ordinary and safe way of therapy from ancient times. The present review mainly concerns the role of the renal KKS in relation to salt-sensitive hypertension. On the basis of above discussion from Section 2 to 6, together with a large volume of supporting evidence, we propose that the reduced activity of the renal KKS plays a crucial role for development of salt-sensitive hypertension and further, maneuvers to enhance the actions of the renal KKS, such as releaser of renal kallikrein and inhibitors of the urinary kininases, CPY and NEP, suppress the development of the salt-sensitive hypertension, at least, in animal models. We already have drugs close to that purpose, such as PNU-37883A for a releaser of renal kallikrein, and, ebelactone B and poststatin for renal kininase inhibitors. We firmly believe that, in the nearest future, these types of the antihypertensive drugs will be developed as drugs in the novel category for salt-sensitive hypertension.

## Abbreviations:

BP: blood pressure
BK: bradykinin
CD: collecting duct
CNT: connecting tubules
CPY: carboxypeptidase Y-like exopeptidase
SBP: systolic blood pressure
